# Curcumol Induces Necroptosis of Hepatic Stellate Cells by Targeting KAT8 to Suppress HK2 Lactylation and Promote HUWE1-Dependent Ubiquitination

**DOI:** 10.7150/ijbs.125009

**Published:** 2026-01-14

**Authors:** Xiaohan Guo, Yuxin Lin, Yue Jiang, Meihui Wang, Yang Li, Yuanyuan Gao, Bocen Chen, Zhengyang Bao, Haoyuan Tian, Xiaomao Chu, Zili Zhang, Jiangjuan Shao, Feng Zhang, Huali Wang, Ji Xuan, Xuefen Xu, Shizhong Zheng

**Affiliations:** 1 Jiangsu Key Laboratory for Pharmacology and Safety Research of Chinese Materia Media, Nanjing University of Chinese Medicine, Nanjing 210023, China.; 2 State Key Laboratory on Technologies for Chinese Medicine Pharmaceutical Process Control and Intelligent Manufacture, Nanjing University of Chinese Medicine, Nanjing 210023, China.; 3 Department of Gastroenterology, Jinling Clinical Medical College, Nanjing University of Chinese Medicine, 305 Zhongshan East Road, Xuanwu Avenue, Nanjing, Jiangsu 210002, China.; 4 Department of General Practice, Nanjing Second Hospital, Nanjing University of Chinese Medicine, Nanjing, Jiangsu 210003, China.

**Keywords:** liver fibrosis, HK2 lactylation, metabolic reprogramming, necroptosis

## Abstract

Liver fibrosis is a pathological outcome of chronic liver injury and is primarily driven by the continuous activation of hepatic stellate cells (HSCs). During activation, HSCs depend on aerobic glycolysis to maintain their fibrogenic characteristics, suggesting that metabolic reprogramming could serve as an effective therapeutic approach. In this study, we demonstrate that Curcumol, a natural compound derived from plants in the Zingiberaceae family, selectively removes activated HSCs by interfering with the lactate-KAT8-HK2 regulatory pathway. In HSCs, lactate produced through glycolysis activates the acetyltransferase KAT8, which catalyzes K346 lactylation of hexokinase 2 (HK2). This modification creates a positive feedback mechanism that stabilizes HK2. Curcumol acts directly on KAT8, inhibiting HK2 lactylation and promoting HUWE1-mediated ubiquitination and degradation of HK2. The resulting loss of HK2 removes its inhibitory influence on RIPK1 ubiquitination, leading to activation of the RIPK1/RIPK3/MLKL signaling pathway and triggering necroptosis. *In vivo* experiments show that Curcumol substantially reduces liver fibrosis, lowers the expression of glycolytic enzymes, and improves liver function in mice with carbon tetrachloride (CCl₄)-induced fibrosis. However, these protective effects are lost when KAT8 is overexpressed. This study highlights HK2 lactylation as a key metabolic control point for HSC survival and identifies Curcumol as a potential anti-fibrotic compound that targets the KAT8-HK2 pathway, linking metabolic inhibition with necroptotic cell death.

## 1. Introduction

Liver fibrosis, the final pathological stage of chronic liver injury caused by factors such as viral infection, alcohol consumption, or metabolic disorders^1^, is a major contributor to liver disease-related deaths worldwide^2^. This process is primarily driven by the persistent activation of hepatic stellate cells (HSCs)^3^. Once activated, HSCs differentiate into myofibroblast-like cells that produce excessive amounts of extracellular matrix (ECM), leading to structural damage and eventual loss of liver function^4^. Although several therapeutic strategies have been proposed to target HSC apoptosis, such as the use of apoptosis inducers like Navitoclax^5^, or to promote ECM degradation through matrix metalloproteinase activators, their clinical application remains limited. These limitations are mainly due to poor targeting specificity and complications such as compensatory fibrosis recurrence^6^. Recent research has shown that activated HSCs undergo distinct metabolic reprogramming, characterized by a reliance on aerobic glycolysis (the Warburg effect) to sustain their pro-fibrotic activity^7^. This metabolic dependency poses a major obstacle to the development of effective anti-fibrotic treatments^8^. The activation of HSCs is closely associated with significant metabolic reprogramming, with glycolytic reprogramming (the Warburg effect) playing a central role in supporting their proliferation and survival^9^. This process is characterized by a shift in energy metabolism, where activated HSCs rely primarily on glycolysis for energy production even in oxygen-rich conditions, while mitochondrial oxidative phosphorylation (OXPHOS) becomes suppressed^8^.

Changes in biosynthetic requirements are also evident, as glycolytic intermediates supply carbon sources essential for collagen production. NADPH is produced through the pentose phosphate pathway to support this process^10^. At the epigenetic regulatory level, glycolytic end products, such as lactate, contribute to histone lactylation, which enhances the transcription of pro-fibrotic genes^11^. Hexokinase 2 (HK2), a key rate-limiting enzyme in glycolysis, is significantly upregulated in activated HSCs^12^. It serves two main functions: catalyzing the phosphorylation of glucose and associating with mitochondria to inhibit apoptotic signaling^13^. Analysis of clinical samples has revealed that HK2 expression is elevated in fibrotic liver tissues and is positively associated with fibrosis severity^14^. These observations indicate that HK2 could be a potential therapeutic target for disrupting the metabolic reliance of HSCs.

Necroptosis (Programmed necrosis) , a regulated type of inflammatory cell death, plays a crucial role in the onset and progression of liver fibrosis^15^. Unlike apoptosis, programmed necrosis is mediated by protein complexes, including RIPK1, RIPK3, and MLKL, leading to membrane disruption and the release of intracellular components^16^. The release of these contents subsequently activates immune responses and promotes inflammation. During liver fibrosis, controlled induction of programmed necrosis in HSCs has been found to effectively eliminate excessively activated HSCs, decrease ECM accumulation, and mitigate fibrotic progression. However, there is currently a lack of targeted therapeutic strategies that can selectively trigger necrosis in HSCs without damaging hepatocytes, representing a major challenge in this area of research.

Curcumol, a bioactive compound derived from plants of the Zingiberaceae family, has emerged as a promising therapeutic candidate. In recent studies, it has demonstrated significant pharmacological effects, including antitumor, antiviral, and antibacterial activities. In the context of liver fibrosis treatment, Curcumol can inhibit the activation of HSCs *in vitro*^17^. However, the precise mechanisms underlying this effect remain unclear. Although some studies indicate that Curcumol may promote apoptosis in HSCs, the specific molecular targets involved in this process have not been fully elucidated^18^. This study aims to determine whether Curcumol can eliminate activated HSCs through non-apoptotic mechanisms, such as necroptosis. This study also aims to explore whether this effect is associated with the regulation of metabolic reprogramming. To date, the specific molecular targets involved in this mechanism and their role in the interplay between cellular metabolism and cell death have not been clearly identified. The findings of this study support the hypothesis that Curcumol exerts a cross-regulatory effect on HSCs by modulating metabolic and cell death signaling pathways. This regulation appears to occur through the suppression of glycolysis, which limits the energy supply to HSCs, and the simultaneous activation of programmed necrosis signaling. These effects contribute to a dual inhibitory mechanism that suppresses HSC activation.

## 2. Results

### 2.1. Multi-omics analysis reveals Curcumol-induced metabolic dysfunction and necroptosis in HSCs

**Figure 1:**
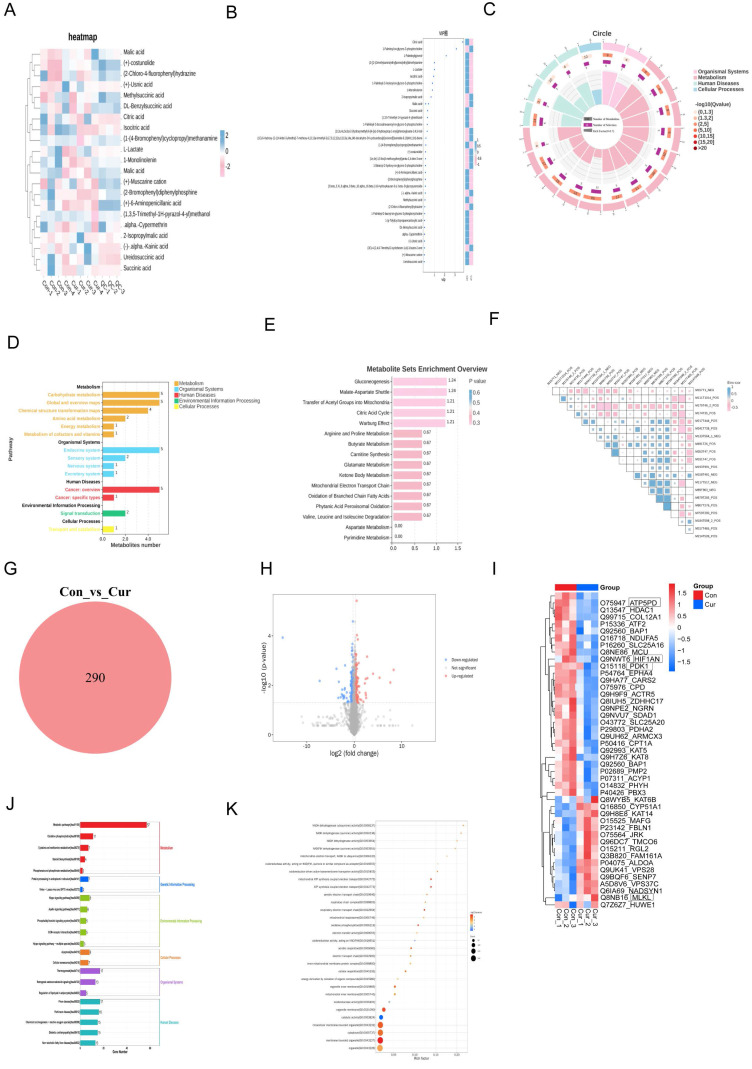
** Multi-omics analysis reveals Curcumol-induced metabolic dysfunction and necroptosis in HSCs.** A. Non-targeted metabolomics analysis of human hepatic stellate cells (LX2) treated with Curcumol showed significant changes in substances related to glycolysis and the tricarboxylic acid cycle, such as lactate, citrate, malate, isocitrate, and succinate, indicating that Curcumol may affect glycolysis and mitochondrial metabolism in HSCs. B. Differential metabolite VIP value plot, with the vertical axis representing metabolites and the horizontal axis representing the VIP values of metabolites. The heatmap on the right shows the expression levels of metabolites across groups. C. Differential metabolite enrichment circle plot. D. KEGG pathway analysis further revealed significant enrichment of metabolism-related pathways, emphasizing the role of glucose metabolism in maintaining cellular energy metabolism balance. E. Differential metabolite pathway enrichment analysis revealed the key role of the glycolytic pathway or Warburg effect in HSCs. F. Differential metabolite correlation enrichment analysis indicated the relationship between metabolic differences and cellular energy metabolism. G. Proteomics of human hepatic stellate cells (LX2) treated with Curcumol, Venn diagram showing the number of differentially expressed proteins detected in the two groups. H. Proteomics of human hepatic stellate cells (LX2) treated with Curcumol, volcano plot showing the distribution of differentially expressed proteins in the two groups. I. Proteomics analysis of human hepatic stellate cells (LX2) treated with Curcumol revealed significant changes in proteins associated with necroptosis, suggesting that Curcumol may induce necroptosis in HSCs. Significant changes were observed in genes and proteins associated with glucose metabolism, mitochondrial oxidative phosphorylation, and fatty acid metabolism. J. KEGG pathway analysis further revealed significant enrichment of cell metabolism-related pathways, emphasizing the role of mitochondrial metabolism in maintaining intracellular energy balance. K. Gene Ontology (GO) analysis indicated that differentially expressed genes were significantly enriched in mitochondrial energy metabolism processes, consistent with mechanisms affecting cellular energy metabolism.

Using proteomics and non-targeted metabolomic analysis of human hepatic stellate cells (LX2) treated with Curcumol, we obtained strong evidence that Curcumol disrupts cellular energy metabolism and induces necroptosis in LX2 cells. The metabolomic data revealed that Curcumol substantially affected several key metabolites involved in glycolysis and the tricarboxylic acid cycle. The analysis of differential metabolites, such as lactate, citrate, and malate, together with the VIP analysis (Fig. 1B), expression heatmap (Fig. 1A), and KEGG pathway enrichment results (Fig. 1D-E), collectively highlight the pivotal role of glycolytic (Warburg effect) and mitochondrial energy metabolism reprogramming in HSCs (Fig. 1A-E). In addition, correlation analysis demonstrates a direct relationship between changes in metabolite profiles and cellular energy imbalance (Fig. 1F). Concurrently, proteomics analysis provided complementary evidence. As shown in Figures 1G and 1H, the Venn diagram and volcano plot quantitatively depict the changes in protein expression profiles. These results revealed notable alterations in proteins involved in glucose metabolism, mitochondrial energy production, and necroptosis (Fig. 1I). In addition, pathway enrichment analysis indicated a significant enrichment of proteins related to mitochondrial OXPHOS and fatty acid metabolism (Fig. 1I and 1J). Gene Ontology (GO) analysis showed that the differentially expressed genes and proteins were predominantly enriched in mitochondrial energy metabolism pathways. As illustrated in Figure 1K, these include processes such as the electron transport chain and ATP synthesis. Based on these findings, we propose that Curcumol may disrupt the coupling between glycolysis and mitochondrial metabolism, resulting in cellular energy collapse. Furthermore, Curcumol may activate the necroptosis pathway to promote the synergistic elimination of HSCs, thereby providing a novel mechanistic insight for its anti-fibrotic effects.

### 2.2. Curcumol induces HSC necroptosis

**Figure 2:**
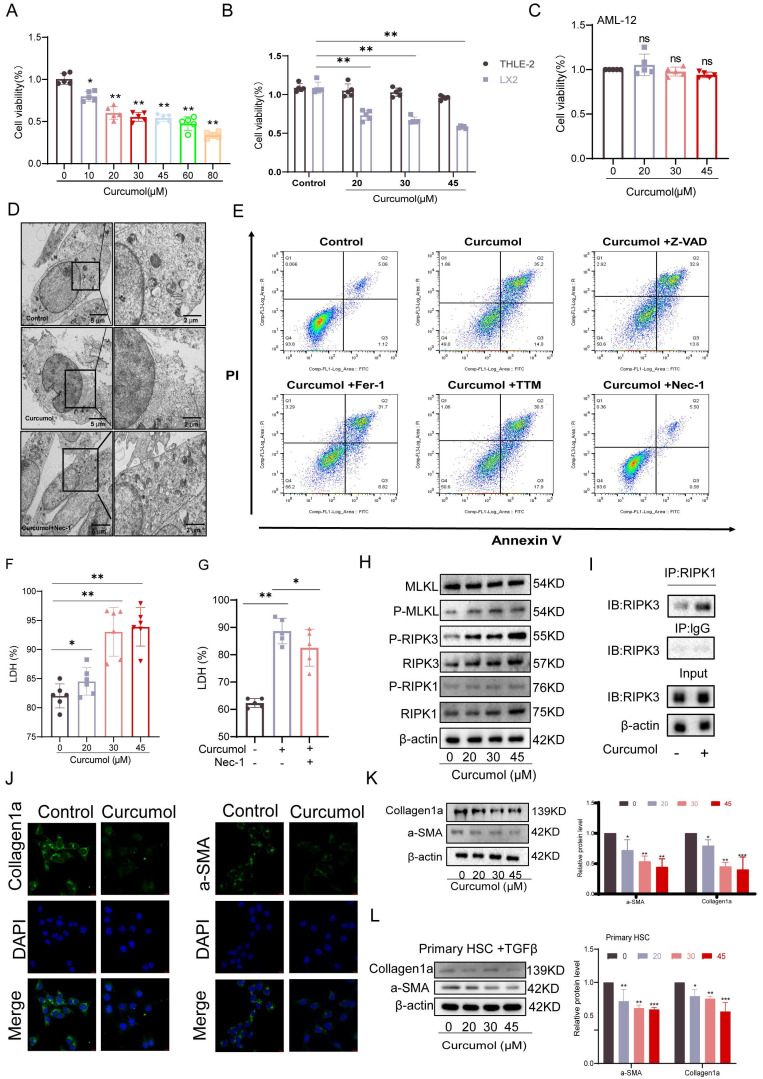
** Curcumol induces HSC necroptosis.** A. LX2 cells were treated with different concentrations of Curcumol (0-80 μM) for 24 h, and cell viability was assessed to determine the optimal intervention concentration (n = 5). B-C. Cell viability was measured in LX2 cells, human normal liver cells Thle-2, and mouse normal liver cells AML12 after treatment with Curcumol (0-45 μM) for 24 h (CCK-8 assay, n = 5). D. LX2 cells were treated with or without Curcumol (30 μM) and the necrotic necroptosis inhibitor Nec-1 (50 μM) for 24 h, and the ultrastructure of the cells was observed using a transmission electron microscope; the right image is an enlarged view of the area framed in the left image (scale bar: 5 μm). E. Under the same treatment conditions, the proportion of necroptotic cells was detected by flow cytometry, and the results were quantitatively analyzed (n = 3). F-G. Detection of LDH release levels in LX2 cells treated with different concentrations of Curcumol (0-45 μM) for 24 h (n = 5); simultaneous comparison of the effects of Nec-1 (50 μM) treatment on Curcumol (30 μM)-induced LDH release, with quantitative analysis (n = 3-5). H. Western blot was used to detect the expression levels of RIPK1, RIPK3, MLKL, and their phosphorylated proteins (p-RIPK1, p-RIPK3, p-MLKL) in LX2 cells after 24 h of treatment with different concentrations of Curcumol (0-45 μM), and quantitative analysis was performed using grayscale analysis (n = 3). I. Co-IP assay to detect the interaction between RIPK1 and RIPK3 in LX2 cells after treatment with Curcumol (30 μM) for 24 h. J. Immunofluorescence assay to detect the expression of Collagen I and α-SMA in LX2 cells after treatment with Curcumol (30 μM) for 24 h (n = 3; scale bar: 10 μm). K-L. Western blotting was used to detect the protein levels of Collagen I and α-SMA in LX2 cells and mouse primary HSCs after 24 h of treatment with different concentrations of Curcumol (0-45 μM), and quantitative analysis was performed using grayscale analysis (n = 3). Data are shown as mean ± SD, and statistical differences were analyzed by one-way ANOVA. ns indicates no significance; *p < 0.05, **p < 0.01, ***p < 0.001.

To identify the optimal concentration of Curcumol for treating LX2 cells, we first assessed its effect on cell viability after 24 h of exposure at varying concentrations (0-80μM). The results indicated that the effective concentration range for further experiments was 0-45μM (Fig. 2A). Subsequent analysis demonstrated that within this range, Curcumol significantly reduced LX2 cell viability after a single 24 h treatment and after prolonged exposure for 48 and 72 h (Fig. 2B, Supplementary Data S1A). In contrast, Curcumol had minimal effects on the viability of normal liver cell types, including human hepatocytes (THLE2) (Fig. 2B, Supplementary Data S1B), human liver sinusoidal endothelial cells (LSEC) (Supplementary Data S1C), mouse hepatocytes (AML12) (Fig. 2C), Primary HSCs of normal mice without TGF-β induction stimulation (Quiescent HSC) (Supplementary Data S1D) and Primary Kupffer cells (KCs) from fibrotic mice (Supplementary Data S1E). These results indicate that Curcumol exerts selective cytotoxic effects on activated HSCs. Along with the observed reduction in cell viability, lactate dehydrogenase (LDH) levels were significantly elevated in Curcumol-treated LX2 cells (Fig. 2F), suggesting loss of membrane integrity and the occurrence of cell death.

To explore the mechanism underlying Curcumol-induced cell death in LX2 cells, we examined the necroptosis pathway based on multi-omics findings and previous studies^6^. Treatment with the necroptosis-specific inhibitor Necrostatin-1 (Nec-1) significantly reversed the morphological alterations associated with Curcumol-induced cell death, as observed under transmission electron microscopy (Fig. 2D). Flow cytometry analysis further demonstrated that Nec-1 significantly decreased the proportion of late apoptotic and necrotic LX2 cells induced by Curcumol(Fig. 2E, Supplementary Data S1H). Furthermore, Nec-1 significantly reduced the Curcumol-induced increase in LDH release (Fig.2G). In contrast, other cell death inhibitors, including the apoptosis inhibitor Z-VAD-FMK (Z-VAD), the ferroptosis inhibitors Ferrostatin-1 (Fer-1) and Liproxstatin-1 (Lipo-1), and the cuproptosis inhibitor Tetrathiomolybdate (TTM), had no notable effect on LDH release caused by Curcumol (Supplementary Data S1F).

At the mechanistic level, western blot analysis revealed that increasing concentrations of Curcumol led to a dose-dependent elevation in the levels of RIPK1, RIPK3, MLKL, and their phosphorylated forms (p-RIPK1, p-RIPK3, and p-MLKL) in LX2 cells (Fig. 2H, Supplementary Data S1G), These results confirm that Curcumol activates the classical RIPK1/RIPK3-mediated necroptotic signaling pathway. Furthermore, co-immunoprecipitation (Co-IP) assays demonstrated that Curcumol treatment enhanced the interaction between RIPK1 and RIPK3 (Fig. 2I), indicating that Curcumol promotes the formation of the necrosome complex, a critical event in the initiation of necroptosis^19^.

The effects of Curcumol on HSC activation markers were evaluated. Immunofluorescence staining and western blot analyses showed that Curcumol treatment significantly reduced the expression of collagen 1 (Collagen1a) and α-smooth muscle actin (α-SMA) in LX2 cells (Fig. 2J,2K), Similar results were confirmed in primary mouse HSCs (Fig. 2L). Curcumol promotes necroptosis in LX2 cells by activating the RIPK1/RIPK3 signaling pathway, facilitating necrosome complex formation, and selectively suppressing HSC activation and proliferation.

### 2.3. Curcumol regulates HK2 function and HSC glycolysis by blocking the lactate-HK2 positive feedback loop

**Figure 3:**
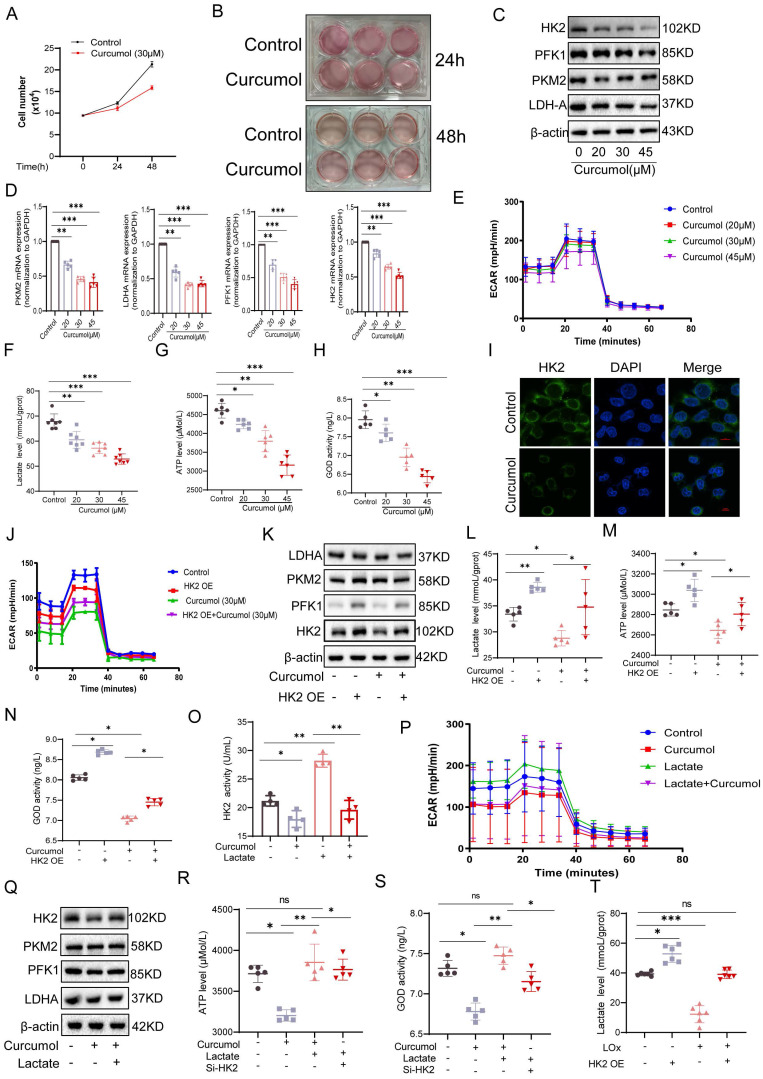
** Curcumol inhibits glycolysis of HSCs by regulating HK2 function through suppression of the lactate-HK2 positive feedback loop.** A. Quantification of LX2 cell numbers treated with vehicle control or Curcumol (30 μM) at the indicated time points (n = 3). B. Representative images showing the color of cell culture medium (n = 3). C. Western blot analysis of HK2, PKM2, PFK1, and LDHA protein expression in LX2 cells treated with Curcumol (0-45 μM) for 24 h, with densitometric quantification (n = 3). D. RT-qPCR analysis of HK2, PKM2, PFK1, and LDHA mRNA expression in LX2 cells treated with Curcumol (0-45 μM) for 24 h (n = 5). E. Seahorse extracellular flux analysis of extracellular acidification rate (ECAR) in LX2 cells treated with Curcumol (0-45 μM) for 24 h (n = 5). F. Lactate levels in LX2 cells treated with Curcumol (0-45 μM) for 24 h, measured using a lactate assay kit (n = 7). G. ATP levels in LX2 cells treated with Curcumol (0-45 μM) for 24 h, determined with an ATP assay kit (n = 5). H. Glucose consumption levels in LX2 cells treated with Curcumol (0-45 μM) for 24 h, determined using a glucose oxidase (GOD) assay kit (n = 5). I. Immunofluorescence staining of HK2 in LX2 cells following Curcumol (30 μM) treatment for 24 h (n = 3; scale bar: 10 μm). J. ECAR changes measured by Seahorse analysis in LX2 cells overexpressing HK2 and treated with Curcumol (30 μM) for 24 h (n = 5). K. Western blot analysis of HK2, PKM2, PFK1, and LDHA protein levels in LX2 cells transfected with an HK2 overexpression plasmid and treated with Curcumol (30 μM) for 24 h, with densitometric quantification (n = 3). L. Lactate levels in LX2 cells transfected with an HK2 overexpression plasmid and treated with Curcumol (30 μM) for 24 h, determined by a lactate assay kit (n = 5). M. ATP levels in LX2 cells transfected with an HK2 overexpression plasmid and treated with Curcumol (30 μM) for 24 h, determined by an ATP assay kit (n = 5). N. Glucose consumption levels in LX2 cells transfected with an HK2 overexpression plasmid and treated with Curcumol (30 μM) for 24 h, assessed with a GOD assay kit (n = 5). O. HK2 enzymatic activity in LX2 cells treated with Curcumol (30 μM) for 24 h in the presence or absence of exogenous sodium lactate (10 mM), measured with an HK2 activity assay kit (n = 5). P. ECAR changes in LX2 cells treated with Curcumol (30 μM) for 24 h in the presence of exogenous sodium lactate (10 mM), determined by Seahorse extracellular flux analysis (n = 5). Q. Western blot analysis of HK2, PKM2, PFK1, and LDHA protein expression in LX2 cells treated with Curcumol (30 μM) for 24 h in the presence of exogenous sodium lactate (10 mM), with densitometric quantification (n = 3). R. ATP levels in LX2 cells transfected with HK2 siRNA and treated with Curcumol (30 μM) and exogenous sodium lactate (10 mM) for 24 h, determined with an ATP assay kit (n = 5). S. Glucose consumption levels in LX2 cells transfected with HK2 siRNA and treated with Curcumol (30 μM) and exogenous sodium lactate (10 mM) for 24 h, assessed with a GOD assay kit (n = 5). T. Lactate levels in LX2 cells transfected with an HK2 overexpression plasmid and treated with the lactate scavenger LOx (2.5 U/mL) for 24 h, determined using a lactate assay kit (n = 5). Data are shown as mean ± SD, and statistical differences were analyzed by one-way ANOVA. ns indicates no significance; *p < 0.05, **p < 0.01, ***p < 0.001.

While examining the mechanism of Curcumol's action on HSCs, we observed that the culture medium of the control group turned yellow more quickly than that of the Curcumol-treated groups (Fig. 3A, B). When considered alongside the metabolomic findings, this observation suggests that Curcumol may interfere with cellular glycolysis. Western blot analysis confirmed that Curcumol treatment led to a concentration-dependent (0-45μM) reduction in the protein expression of key glycolytic enzymes, including HK2, PKM2, PFK1, and LDHA, in HSCs (Fig. 3C, Supplementary Data S3A). In contrast, Curcumol did not significantly alter the protein expression of these glycolytic enzymes or lactate production in quiescent primary mouse HSCs (Supplementary Data S2A), LSEC (Supplementary Data S2B), and primary Kupffer cells from fibrotic mice (Supplementary Data S2C), Curcumol did not significantly affect the protein levels of these glycolytic enzymes or lactate production (Supplementary Data S2D). Moreover, quantitative real-time PCR (qPCR) analysis showed that Curcumol modulated HK2 mRNA expression (Fig. 3D), indicating that its effects on glycolysis in HSCs may occur through regulation of HK2. To further assess glycolytic activity in LX2 cells, metabolic profiling was conducted using the Seahorse XF Analyzer. The results demonstrated that Curcumol treatment caused a dose-dependent reduction in the extracellular acidification rate (ECAR) (Fig. 3E), along with decreased lactate production (Fig. 3F), lower ATP levels (Fig. 3G), and reduced glucose consumption (Fig. 3H). These results indicate that Curcumol significantly reduces the glycolytic capacity of LX2 cells by downregulating the expression and activity of key glycolytic enzymes. Among these, HK2 appears to be the most affected target. Previous studies have shown that both HSC-specific and systemic HK2 deficiency suppress HSC activation and attenuate liver fibrosis *in vivo*^20^. Therefore, it is plausible that Curcumol primarily modulates glycolysis in HSC through its regulatory effect on HK2. Immunofluorescence analysis showed that Curcumol treatment decreased HK2 expression in HSCs (Fig. 3I), further validating that Curcumol regulates HSC glycolysis by suppressing HK2. Using an LX2 cell model with HK2 overexpression (Supplementary Data S3B), we found that after Curcumol treatment, the ECAR in HK2-overexpressing cells remained comparable to that of the control group (Fig. 3J). Furthermore, HK2 overexpression significantly reversed the inhibitory effects of Curcumol on several glycolytic indicators, including glycolytic enzyme expression (Fig. 3K, Supplementary Data S3D), lactate production (Fig. 3L), ATP synthesis (Fig. 3M), and and glucose consumption (Fig. 3N). These results reveal that Curcumol primarily decreases glycolytic flux in HSCs by inhibiting HK2 protein function. The finding that HK2 overexpression reverses this effect further confirms that Curcumol regulates metabolic reprogramming in HSCs primarily through functional inhibition of HK2. After establishing that Curcumol suppresses glycolysis in HSCs by targeting HK2, we further investigated the upstream regulatory mechanisms. Activated HSCs exhibit increased glycolytic activity, elevated lactate accumulation, and abnormally high HK2 expression. An increasing number of studies have revealed that lactate functions not only as a metabolic byproduct but also as a signaling molecule that can modulate diverse protein activities. However, whether lactate exerts a direct regulatory effect on HK2 remains uncertain.

In this study, we observed that the addition of exogenous lactate effectively mitigated the inhibitory effect of 30 μM Curcumol on HK2 activity (Fig. 3O) and restored the expression of key glycolytic enzymes (Fig. 3Q, Supplementary Data S3E). Furthermore, lactate supplementation reversed the Curcumol-induced reductions in ECAR (Fig. 3P), ATP production (Fig. 3R) and glucose consumption (Fig. 3S). To further confirm the role of lactate, intracellular lactate was degraded using lactate oxidase (LOx). This treatment led to a pronounced decrease in intracellular lactate levels in HSCs and effectively inhibited the lactate accumulation induced by HK2 overexpression (Fig. 3T). Collectively, these findings indicate that lactate is essential for maintaining HK2 function and glycolytic activity in HSCs, implying that lactate serves as a key metabolic signaling mediator in the metabolic reprogramming associated with HSC activation.

### 2.4. Curcumol Inhibits HK2 K346 lactylation, blocking its nuclear translocation and inducing mitochondrial dysfunction in HSCs

**Figure 4:**
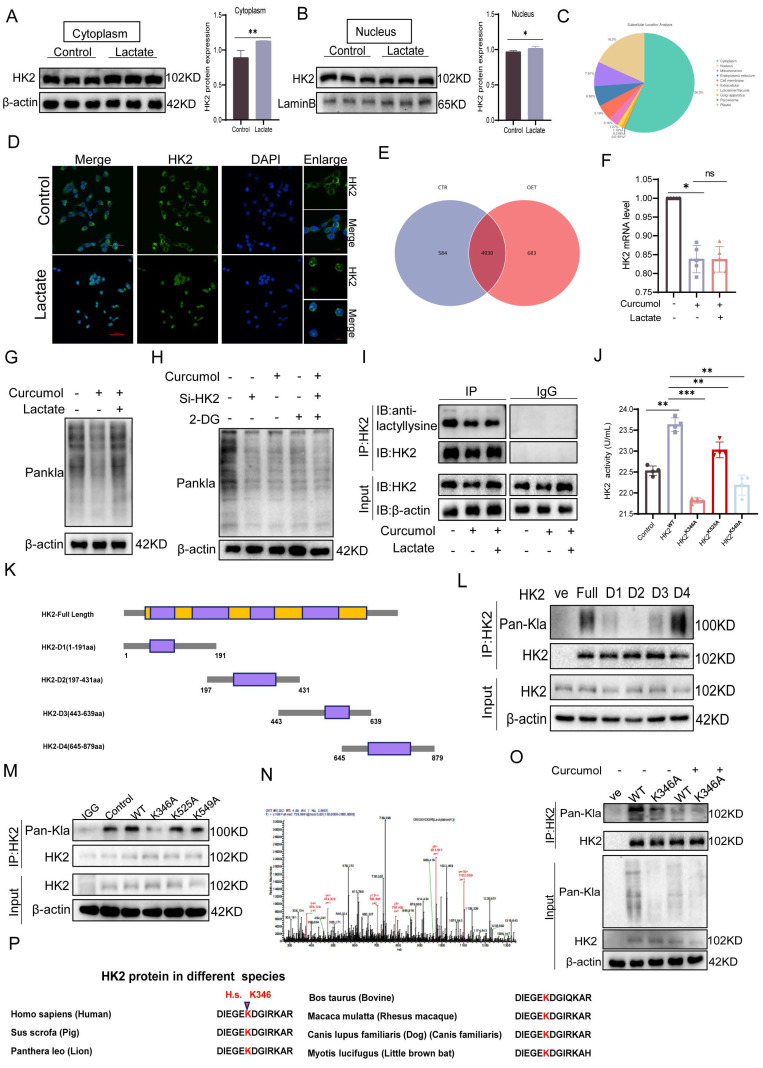

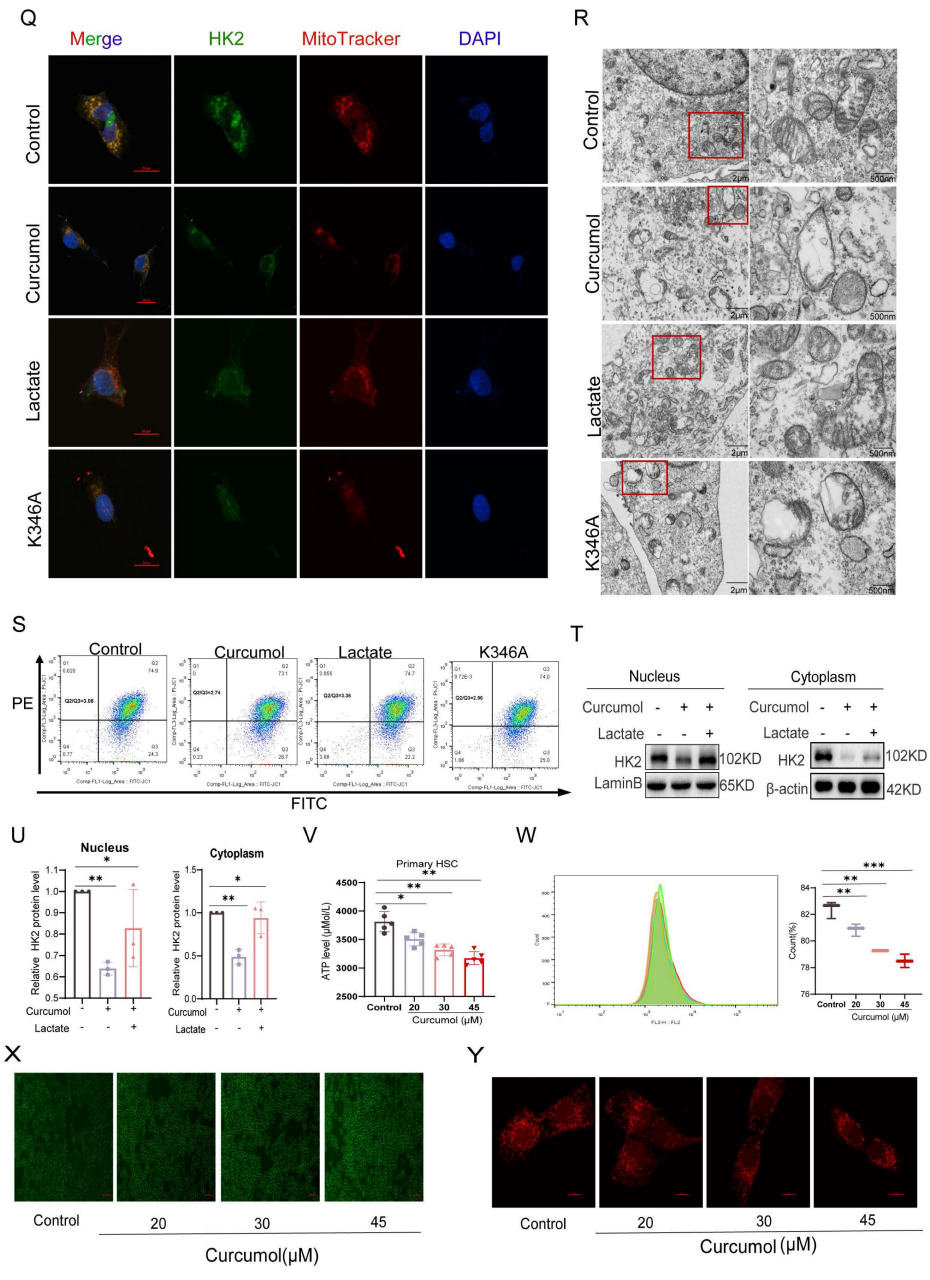
** Curcumol blocks HK2 nuclear translocation and induces mitochondrial dysfunction in HSCs by inhibiting HK2 K346 lactylation.** A-B. Western blot analysis of HK2 expression in cytoplasmic and nuclear fractions of LX2 cells treated with sodium lactate (10 mM) for 24 h, with densitometric quantification (n = 3). C,E. IP-MS analysis of differentially expressed proteins in sodium lactate-treated LX2 cells, followed by subcellular localization prediction using DeepLoc2 (n = 3). D. Immunofluorescence staining of HK2 in LX2 cells treated with sodium lactate (10 mM) for 24 h (n = 3; scale bar: 50 μm, 10 μm). F. qPCR analysis of HK2 mRNA levels in LX2 cells treated with sodium lactate (10 mM) and Curcumol (30 μM) for 24 h (n = 5). G. Western blot analysis of global protein lactylation (Pan-Kla) in LX2 cells pretreated with or without sodium lactate (10 mM) followed by Curcumol (30 μM) for 24 h (n = 3). H. Western blot detection of Pan-Kla levels in LX2 cells transfected with HK2 siRNA and treated with Curcumol (30 μM) plus 2-DG (5 mM) (n = 3). I. Immunoprecipitation assay confirming lactylation of HK2 protein in LX2 cells. J.HK2 enzymatic activity in LX2 cells transfected with HK2-WT, HK2-K346A, HK2-K525A, or HK2-K549A plasmids, measured with an HK2 activity assay. K. Schematic diagram of HK2 truncation plasmid constructs. L-M. Co-immunoprecipitation (Co-IP) analysis of HK2 lactylation in LX2 cells transfected with HK2-WT, truncation mutants (D1-D4), or site-directed mutants (K346A, K525A, K549A) (n = 3). N. IP-MS analysis of PKM2-associated HK2 in sodium lactate-treated LX2 cells, identifying potential lactylation sites (n = 3). O. IP analysis of HK2 lactylation in LX2 cells transfected with HK2-WT or HK2-K346A plasmids and treated with or without Curcumol (30 μM) for 24 h. P. Sequence alignment showing evolutionary conservation of the HK2 K346 site; lysine residues corresponding to human HK2 K346 are highlighted in red. Q. Immunofluorescence analysis of HK2 mitochondrial co-localization in LX2 cells transfected with HK2-K346A and treated with Curcumol (30 μM) plus sodium lactate (10 mM) for 24 h (n = 3). R. Transmission electron microscopy images of LX2 cells transfected with HK2-K346A and treated with Curcumol (30 μM) plus sodium lactate (10 mM) for 24 h (right: magnified boxed region; scale bars: left = 5 μm, right = 500 nm). S. Under the same treatment conditions, the proportion of necroptotic cells was detected by flow cytometry, and the results were quantitatively analyzed (n = 3). T-U. Western blot analysis of HK2 protein distribution in cytoplasmic and nuclear fractions of LX2 cells treated with Curcumol (30 μM) and sodium lactate (10 mM) for 24 h, with quantification (n = 3). V.ATP levels in primary mouse HSCs treated with Curcumol (0-45 μM) for 24 h, measured with an ATP assay (n = 5). W. Flow cytometry analysis of mitochondrial membrane potential in LX2 cells treated with Curcumol (0-45 μM) for 24 h (n = 3). X. Intracellular ROS levels in LX2 cells treated with Curcumol (0-45 μM) for 24 h, measured by fluorescent probe staining (scale bar: 100 μm). Y. Mitochondrial content in LX2 cells treated with Curcumol (0-45 μM) for 24 h, detected using a mitochondrial-specific fluorescent probe (scale bar: 10 μm). Data are shown as mean ± SD, and statistical differences were analyzed by one-way ANOVA. ns indicates no significance; *p < 0.05, **p < 0.01, ***p < 0.001.

To further determine whether Curcumol modulates HK2 through lactate-mediated post-translational modification, a series of experiments was conducted. Initially, treatment with exogenous sodium lactate markedly enhanced the nuclear translocation of HK2(Fig. 4D) and elevated its cytoplasmic protein levels (Fig. 4A-B, Supplementary Data S3F), while no significant change was observed in HK2 mRNA expression (Fig. 4F). These findings indicate that lactate modulates HK2 through post-translational mechanisms rather than transcriptional regulation. Furthermore, IP-MS analysis revealed that lactoylated proteins (Fig. 4E) were predominantly localized in the cytoplasm, with additional enrichment observed in the nucleus and mitochondria (Fig. 4C). Western blot analysis revealed that Curcumol treatment decreased the overall level of protein lactoylation in cells, whereas supplementation with exogenous lactate partially restored this modification (Fig. 4G). Moreover, combining Curcumol with the glycolysis inhibitor 2-DG and HK2 siRNA further suppressed protein lactylation (Fig. 4H). Co-IP experiments provided direct evidence that Curcumol significantly inhibited the lactylation modification of HK2 (Fig. 4I).

To identify the specific modification site, HK2 truncation mutants (D1-D4) were generated, which localized lactylation to the D2 domain (Fig. 4K-L). Subsequent site-directed mutagenesis revealed K346 as the key lactylation site (Fig. 4M-N), whereas mutations at K525A and K549A had minimal impact. Mutation of K346 not only substantially decreased HK2 lactylation but also impaired its enzymatic activity (Fig. 4J). Sequence alignment showed that this site is highly conserved across species (Fig. 4P). Importantly, Curcumol inhibited lactylation of wild-type HK2 (HK2-WT) but had little effect on the K346A mutant (Fig. 4O), indicating that Curcumol specifically targets HK2 lactylation at K346. Immunofluorescence analysis further confirmed that Curcumol reduced HK2 localization in both mitochondria and the nucleus, which was supported by subcellular fractionation experiments (Fig. 4Q). Lactate supplementation completely restored HK2 nuclear translocation, whereas the K346A mutant reproduced the effects of Curcumol, suggesting that lactylation at K346 serves as a molecular switch controlling HK2 subcellular localization. At the organelle level, transmission electron microscopy revealed extensive mitochondrial damage following Curcumol treatment, including disrupted cristae and swollen matrices (Fig. 4R). Although lactate supplementation or HK2 K346 mutation did not reverse these structural abnormalities, they partially mitigated the Curcumol-induced loss of mitochondrial membrane potential (Fig. 4S). Although lactate restored HK2 localization, it did not reverse the structural damage, indicating that lactylation influences mitochondrial integrity beyond regulating subcellular localization. Additional experiments demonstrated that Curcumol significantly increased mitochondrial ROS levels (Fig. 4X), altered HK2 distribution between the cytoplasm and nucleus (Fig. 4T-U), decreased ATP production (Fig. 4V), reduced mitochondrial abundance (Fig. 4Y), and disrupted mitochondrial membrane potential (Fig. 4W). In summary, Curcumol selectively inhibits HK2 lactylation at the K346 site, preventing its nuclear translocation and mitochondrial localization. This disruption results in mitochondrial dysfunction and oxidative stress, ultimately destabilizing the homeostasis of activated HSCs.

### 2.5. Curcumol induces HSCs necroptosis by suppressing RIPK1 ubiquitination via HK2

**Figure 5:**
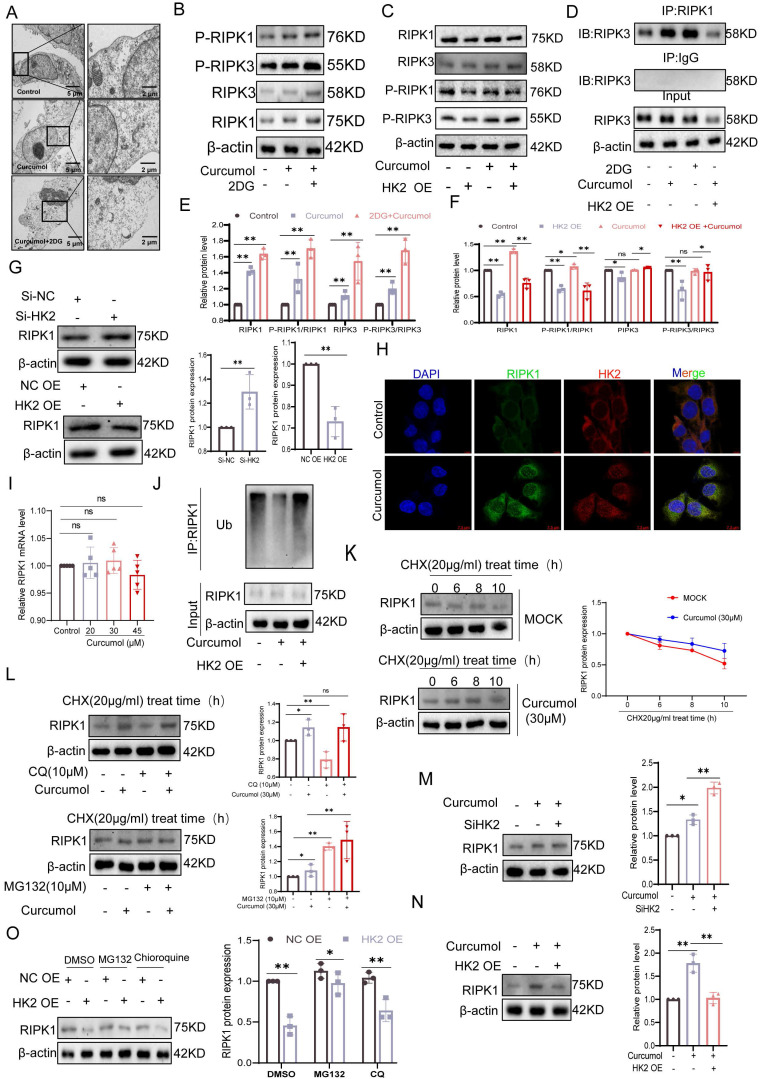
** Curcumol induces HSCs necroptosis by suppressing HK2-mediated ubiquitination of RIPK1.** A. Transmission electron microscopy images of LX2 cells treated with Curcumol (30 μM) for 24 h in the presence or absence of 2-DG (5 mM) (right: magnified region; scale bar: 5 μm). B, E. Western blot analysis of RIPK1, RIPK3, p-RIPK1, and p-RIPK3 expression in LX2 cells treated with Curcumol (30 μM) and 2-DG (5 mM) for 24 h, with densitometric quantification (n = 3). C, F. Western blot analysis of RIPK1, RIPK3, p-RIPK1, and p-RIPK3 expression in LX2 cells transfected with HK2 overexpression plasmid and treated with Curcumol (30 μM) for 24 h, with densitometric quantification (n = 3). D. Co-immunoprecipitation (Co-IP) analysis of RIPK1-RIPK3 interaction in LX2 cells transfected with HK2 overexpression plasmid and treated with Curcumol (30 μM) plus 2-DG (5 mM) for 24 h. G. Western blot analysis of RIPK1 expression in HK2 siRNA-transfected HK2-overexpressing LX2 cells, with densitometric quantification (n = 3). H. Immunofluorescence staining showing colocalization of HK2 and RIPK1 in LX2 cells treated with Curcumol (30 μM) for 24 h (n = 3; scale bar: 2.5 μm). I. RT-qPCR analysis of RIPK1 mRNA expression in LX2 cells treated with increasing concentrations of Curcumol (0-45 μM) for 24 h (n = 5). J. Immunoprecipitation analysis of RIPK1 ubiquitination in LX2 cells transfected with HK2 overexpression plasmid and treated with Curcumol (30 μM) for 24 h (n = 3). K. Western blot analysis of RIPK1 protein stability in LX2 cells treated with Curcumol (30 μM), cycloheximide (CHX, 20 μg/mL) for 24 h in the presence or absence of protein synthesis inhibitor, with quantification of half-life (n = 3). L. Western blot analysis of RIPK1 protein expression in LX2 cells treated with Curcumol (30 μM), cycloheximide (CHX, 20 μg/mL), proteasome inhibitor MG132 (10 μM), alone or in combination, with quantification (n = 3). M,N. Western blot analysis of RIPK1 protein levels in LX2 cells co-transfected with HK2 siRNA and HK2 overexpression plasmid, followed by Curcumol (30 μM) treatment for 24 h, with quantification (n = 3). O. Western blot analysis of RIPK1 expression in LX2 cells transfected with HK2 overexpression plasmid and treated with autophagy inhibitor chloroquine (CQ, 20 μg/mL), MG132 (10 μM), alone or in combination, for 24 h, with quantification (n = 3). Data are shown as mean ± SD, and statistical differences were analyzed by one-way ANOVA. ns indicates no significance; *p < 0.05, **p < 0.01, ***p < 0.001.

To assess the impact of Curcumol on necroptosis in LX2 cells and the involvement of HK2, transmission electron microscopy showed that 24-hour Curcumol treatment induced classical necrotic features, including plasma membrane rupture and organelle swelling (Fig. 5A). This effect was significantly enhanced by the glycolysis inhibitor 2DG, indicating a synergistic interaction between metabolic stress and necroptotic signaling. At the molecular level, Curcumol specifically activated the RIPK1/RIPK3 phosphorylation cascade, while HK2 overexpression completely prevented this activation (Fig. 5B-C,5E-F), confirming HK2 as a critical checkpoint in necroptosis.

In HK2-overexpressing LX2 cells, co-immunoprecipitation assays showed that HK2 facilitated the interaction between RIPK1 and RIPK3(Fig. 5D). Western blot analysis further indicated that HK2 overexpression significantly decreased RIPK1 protein levels, whereas HK2 knockdown using siRNA partially restored RIPK1 expression (Fig. 5G). To exclude transcriptional regulation, we examined changes in RIPK1 mRNA levels following either overexpression or knockdown of HK2. The results showed that altering HK2 expression did not significantly affect RIPK1 mRNA levels (Supplementary Fig. S3K), further demonstrating that HK2-mediated changes in RIPK1 expression occur primarily at the post-translational level. Consistently, immunofluorescence analysis demonstrated increased intracellular co-localization of HK2 and RIPK1 following Curcumol treatment (Fig. 5H). Considering the key role of ubiquitination in controlling protein stability, we hypothesized that Curcumol may influence RIPK1 ubiquitination via HK2, thereby affecting its stability. Immunoprecipitation experiments supported this hypothesis, showing that RIPK1 ubiquitination was markedly reduced in Curcumol-treated LX2 cells overexpressing HK2 (Fig. 5J). To further explore the specific role of HK2 in this process, we generated an HK2 K346 site mutant and conducted ubiquitination analysis. The results demonstrated that mutation at K346 effectively suppressed RIPK1 ubiquitination (Supplementary Data S3G), identifying K346 as a critical site for HK2-mediated regulation of RIPK1 ubiquitination.

RT-qPCR analysis excluded transcriptional regulation, as Curcumol treatment did not significantly alter RIPK1 mRNA levels (Fig. 5I). Protein stability experiments revealed that Curcumol markedly prolonged the half-life of RIPK1 (Fig. 5K). Furthermore, cycloheximide (CHX) chase experiments and treatment with the proteasome inhibitor MG132 confirmed that RIPK1 stability is controlled via the ubiquitin-proteasome pathway (Fig. 5L). Consistently, co-treatment of HSCs with the autophagy inhibitor chloroquine (CQ) and curcumol revealed that autophagy inhibition did not alter the upregulation of RIPK1 protein levels induced by curcumol (Supplementary Fig. S6A, S6D). Furthermore, assessment of autophagy flux markers LC3-II/I and p62 (Supplementary Fig. S6A-C) indicated that curcumol had no significant effect on basal autophagy in HSCs. These findings further support the conclusion that HK2 regulates RIPK1 through the ubiquitin-proteasome pathway. Notably, this regulation was dependent on HK2: HK2 knockdown significantly decreased RIPK1 protein levels (Fig. 5M), whereas HK2 overexpression counteracted Curcumol's effect on RIPK1 degradation (Fig. 5N-O). Collectively, these results indicate that Curcumol stabilizes RIPK1 by suppressing its ubiquitination through HK2, thereby activating necroptotic signaling and inducing necroptosis in LX2 cells.

### 2.6. KAT8 mediates HK2 lactylation at K346

**Figure 6:**
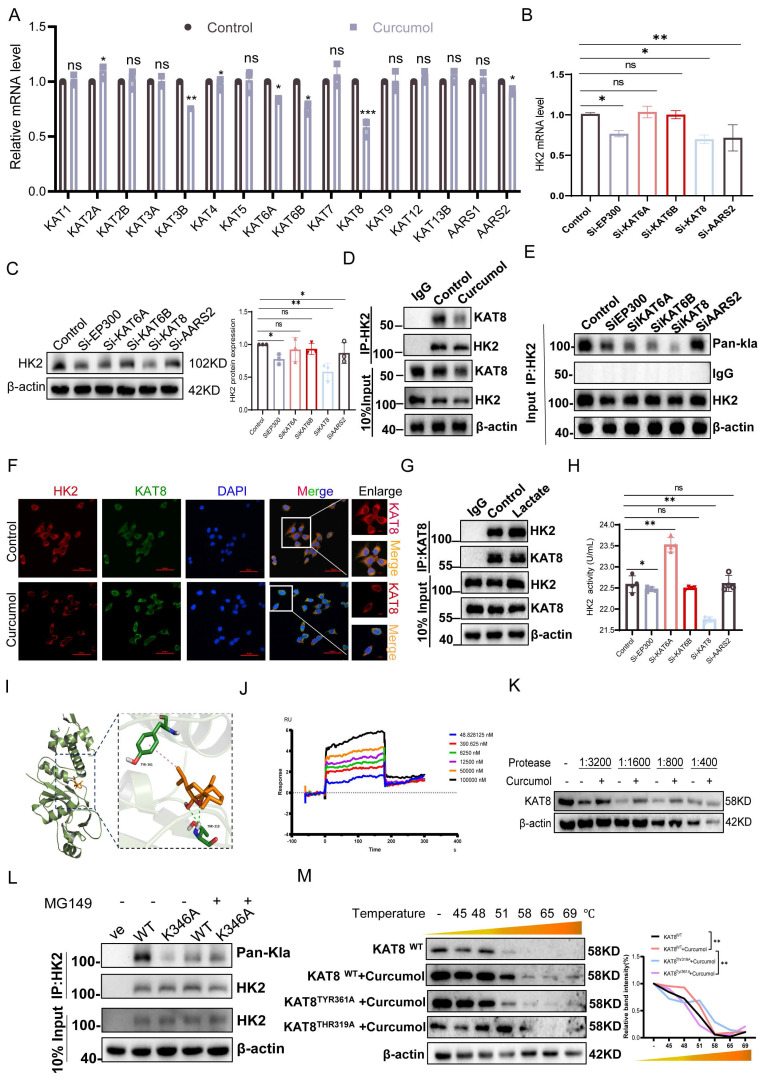
** KAT8 mediates lactylation modification of HK2 at the K346 site.** A. RT-qPCR analysis of mRNA expression of key lactylation-modifying enzymes in LX2 cells treated with Curcumol (30 μM) for 24 h (n = 5). B. RT-qPCR analysis of HK2 mRNA expression in LX2 cells transfected with siRNAs targeting EP300, KAT6A, KAT6B, KAT8, or AARS2 (n = 5). C. Western blot analysis of HK2 protein levels in LX2 cells following transfection with EP300, KAT6A, KAT6B, KAT8, or AARS2 siRNAs (n = 3). D. Co-immunoprecipitation (Co-IP) analysis of HK2-KAT8 interaction in LX2 cells treated with Curcumol (30 μM) for 24 h. E. Co-IP analysis of HK2 lactylation levels in LX2 cells following siRNA-mediated knockdown of EP300, KAT6A, KAT6B, KAT8, or AARS2. F. Immunofluorescence staining showing colocalization of HK2 and KAT8 in LX2 cells treated with Curcumol (30 μM) for 24 h (n = 3; scale bar: 50 μm). G. Co-IP analysis of HK2-KAT8 interaction in LX2 cells treated with or without exogenous sodium lactate (10 mM). H. Enzymatic activity assay of HK2 in LX2 cells transfected with siRNAs against EP300, KAT6A, KAT6B, KAT8, or AARS2 (n=4). I. Molecular docking simulation predicting potential binding sites of Curcumol on KAT8, focusing on TYR-361 and THR-319 residues. J. Surface plasmon resonance (SPR) was used to assess the binding affinity between Curcumol and purified KAT8 protein (n=3). K.LX2 cells were treated with Curcumol (30 μM) and then digested with various concentrations of protease. Subsequently, KAT8 protein levels were determined by western blot. L. Western blot analysis of HK2 lactylation levels in LX2 cells transfected with HK2-WT or HK2-K346A plasmids and treated with the KAT8 inhibitor MG149 (50μM). M. Cellular thermal shift assay (CETSA) assessing the effect of Curcumol (30 μM, 24 h) on binding stability of wild-type KAT8 or mutant KAT8-TYR361A and KAT8-THR319A in LX2 cells, with emphasis on TYR361/THR319 residues; *p < 0.05, **p < 0.01, ***p < 0.001.

To investigate the molecular mechanism by which Curcumol modulates HK2 lactylation, we performed high-throughput RT-qPCR to examine its effects on the expression of lactylation-related enzymes in LX2 cells. After 24 h of treatment, the mRNA levels of KAT3B (EP300), KAT6A, KAT6B, KAT8, and AARS2 were significantly reduced (Fig. 6A). To identify key regulatory factors, we conducted siRNA-mediated knockdown experiments (Supplementary Data S3H-J). The findings indicated that silencing KAT8 significantly decreased both mRNA (Fig. 6B) and protein (Fig. 6C) expression levels of HK2, whereas knockdown of the other candidate genes exhibited minimal effects. These findings indicate that KAT8 plays a specific and critical role in regulating HK2 expression. At the post-translational level, KAT8 knockdown markedly reduced HK2 lactylation, whereas knockdown of other siRNA groups exhibited minimal effects (Fig. 6E). Similarly, treatment with the KAT8-specific inhibitor (MG149) suppressed HK2 lactylation, an effect that was completely absent in the HK2 K346 mutant (Fig. 6L), directly confirming KAT8 as the enzyme responsible for catalyzing lactylation at this site. Co-immunoprecipitation experiments further demonstrated that Curcumol treatment weakened the interaction between KAT8 and HK2 (Fig. 6D). Immunofluorescence analysis further confirmed their intracellular co-localization, which was disrupted following Curcumol treatment (Fig. 6F).

The addition of exogenous lactate strengthened the interaction between KAT8 and HK2 (Fig. 6G), suggesting that lactate may function as a signaling molecule to promote their association and facilitate HK2 lactylation. Enzymatic activity assays demonstrated that KAT8 knockdown significantly decreased HK2 activity, whereas knockdown of other siRNA groups had minimal impact (Fig. 6H). To investigate whether Curcumol directly targets KAT8, we first performed molecular docking analysis, which indicated a potential interaction between Curcumol and KAT8 (Fig. 6I). We then employed the Drug Affinity Responsive Target Stability (DARTS) assay, which relies on the principle that a target protein becomes less susceptible to proteolysis upon drug binding. Our results showed that Curcumol treatment significantly stabilized KAT8 against proteolytic degradation, providing direct biochemical evidence for their interaction (Fig. 6K, Supplementary Data S5H). Furthermore, Surface Plasmon Resonance (SPR) analysis quantitatively confirmed the binding, revealing a high-affinity interaction between Curcumol and immobilized KAT8 (Fig. 6J). Building on this confirmed direct binding, we conducted Cellular Thermal Shift Assay (CETSA) experiments to map the precise binding site, which revealed that the TYR361A and THR319A mutants exhibited significantly lower binding affinity for Curcumol compared with wild-type KAT8 (Fig. 6M). Finally, Curcumol treatment increased the interaction between KAT8 and HK2 (Supplementary Data S5I). Collectively, these results demonstrate that KAT8 directly catalyzes the lactylation of HK2 at K346, whereas Curcumol directly binds to KAT8 to inhibit this modification, thereby disrupting HK2-mediated metabolic reprogramming.

### 2.7. Curcumol promotes HUWE1-dependent ubiquitination and degradation of HK2 by inhibiting KAT8-mediated K346 lactylation

**Figure 7:**
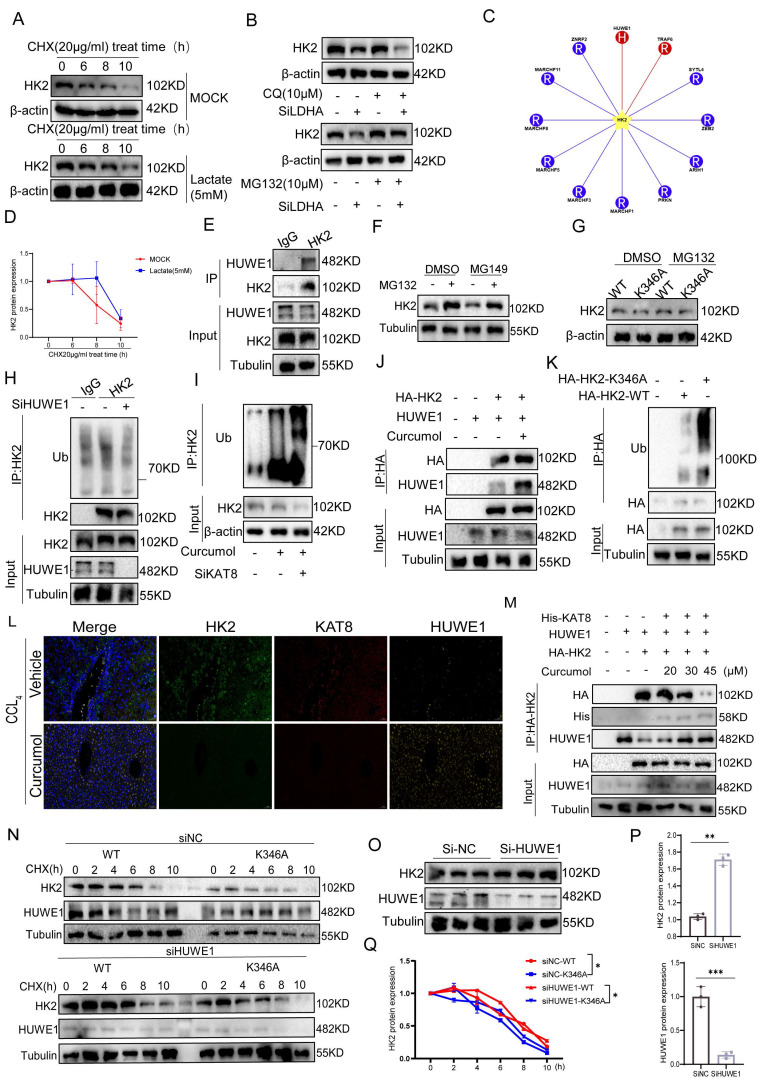
** Curcumol promotes HUWE1-dependent ubiquitin-mediated degradation of HK2 by inhibiting KAT8-mediated lactylation at the K346 site.** A, D. Western blot analysis of HK2 protein half-life in LX2 cells treated with or without protein synthesis inhibitors in the presence of sodium lactate (10 mM, 24 h), with quantification of band intensity (n = 3). B. Western blot analysis of HK2 protein levels in LX2 cells transfected with LDHA siRNA or control siRNA and treated with chloroquine (CQ, 20 μg/mL), MG132 (10 μM), alone or in combination for 24 h (n = 3). C. Schematic diagram illustrating regulators of HK2 ubiquitination. E. Co-immunoprecipitation (Co-IP) analysis of the interaction between HK2 and the E3 ubiquitin ligase HUWE1. F. Western blot analysis of HK2 protein levels in LX2 cells treated with MG149 (50 μM, 48 h), with or without MG132 (10 μM, 6 h), followed by densitometric quantification (n = 3). G. Western blot analysis of HK2 protein levels in LX2 cells expressing HK2-WT or HK2-K346A after treatment with MG132 (10 μM, 8 h) (n = 3). H. Co-IP analysis of HK2 ubiquitination in LX2 cells with or without HUWE1 siRNA transfection (24 h). I. Co-IP analysis of HK2 ubiquitination in LX2 cells transfected with or without KAT8 siRNA (24 h) and treated with Curcumol (30 μM). J. Co-IP analysis was used to examine the interaction between HK2 and HUWE1 in LX2 cells that were co-transfected with the indicated plasmids and treated with Curcumol (30 μM, 8 h). K. Co-IP analysis of HK2 ubiquitination in LX2 cells expressing HK2-WT or HK2-K346A. L. Immunofluorescence analysis of HK2 (green), KAT8 (red), and HUWE1 (yellow) colocalization in liver tissues from CCl₄-induced fibrotic mice and Curcumol-treated mice (n = 3; scale bar: 50 μm). M. Co-IP analysis was performed on the co-transfected plasmids for 24 hours, and then the interactions between HK2, KAT8, and HUWE1 in LX2 cells treated with different concentrations of Curcumol (0, 20, 30, 45 μM) were examined. N, Q. Western blot analysis of HK2 degradation kinetics in LX2 cells expressing HK2-WT or HK2-K346A following HUWE1 siRNA transfection (24 h) and cycloheximide (CHX, 20 μg/mL) treatment for the indicated times (n = 3). O, P. Western blot analysis of HK2 protein levels in LX2 cells transfected with HUWE1 siRNA (24 h), with densitometric quantification (n = 3). Data are shown as mean ± SD, and statistical differences were analyzed by one-way ANOVA. ns indicates no significance; *p < 0.05, **p < 0.01, ***p < 0.001.

To explore the role of lactate in regulating HK2 protein stability, we first conducted protein half-life assays. Treatment of LX2 cells with 5 mM lactate for 24 h significantly slowed HK2 degradation (Fig. 7A). Under conditions of inhibited protein synthesis, density analysis confirmed that the half-life of HK2 was significantly extended (Fig. 7D), indicating that lactate stabilizes HK2 by suppressing protein degradation. Further experiments revealed that LDHA knockdown (Supplementary Data S4A) significantly decreased HK2 protein levels. This decrease was fully rescued by the proteasome inhibitor MG132, whereas the autophagy inhibitor CQ had no effect (Fig. 7B, Supplementary Data S4B), indicating that HK2 degradation is proteasome-dependent and requires LDHA-mediated lactate production. Through E3 ubiquitinase screening, HUWE1 was identified as the primary ligase responsible for HK2 ubiquitination (Fig. 7C,7H), and Co-IP experiments confirmed a direct interaction between HK2 and HUWE1 (Fig. 7E). Further analysis showed that the inhibition of the lactylation enzyme KAT8 using MG149 substantially increased HK2 protein levels, while treatment with the proteasome inhibitor MG132 blocked the HK2 degradation induced by KAT8 inhibition (Fig. 7F, Supplementary Data S4C). Moreover, Curcumol treatment and KAT8 knockdown increased HK2 ubiquitination, exhibiting a synergistic effect (Fig. 7I), suggesting that Curcumol may promote HUWE1-mediated ubiquitination by inhibiting KAT8-dependent lactylation. Under proteasome inhibition, wild-type HK2 (WT) accumulated substantially, whereas the K346A mutant did not show significant accumulation (Fig. 7G, Supplementary Data S4D). Ubiquitination assays showed that the K346A mutant exhibited significantly higher ubiquitination levels than WT HK2 (Fig. 7K). Notably, HUWE1 knockdown substantially increased the stability of WT HK2 (Fig. 7O-P) but did not affect the K346A mutant. In HUWE1-deficient cells, the K346A mutant continued to undergo rapid degradation (Fig. 7N,7Q). These findings indicate a competitive association between KAT8-mediated K346 lactylation and HUWE1-driven ubiquitination in controlling HK2 stability.

Overexpression experiments in HEK293T cells revealed that KAT8 overexpression disrupted the interaction between HUWE1 and HK2 (Fig. 7M), Curcumol promotes their interaction (Fig. 7J). Immunofluorescence analysis demonstrated co-localization of HK2, KAT8, and HUWE1 in liver tissues, with HK2-KAT8 co-localization significantly enhanced in fibrotic mouse livers (Fig. 7L), indicating activation of this regulatory pathway under pathological conditions. Collectively, these findings indicate that lactate activates KAT8 to catalyze HK2 K346 lactylation, which impairs HUWE1-mediated ubiquitination and prevents proteasomal degradation of HK2. Curcumol counteracts this effect by inhibiting KAT8-mediated lactylation, restoring HUWE1 activity, and promoting ubiquitin-dependent HK2 degradation.

### 2.8. Curcumol targets KAT8 to inhibit HSC glycolysis and induce necroptosis *in vivo*

**Figure 8:**
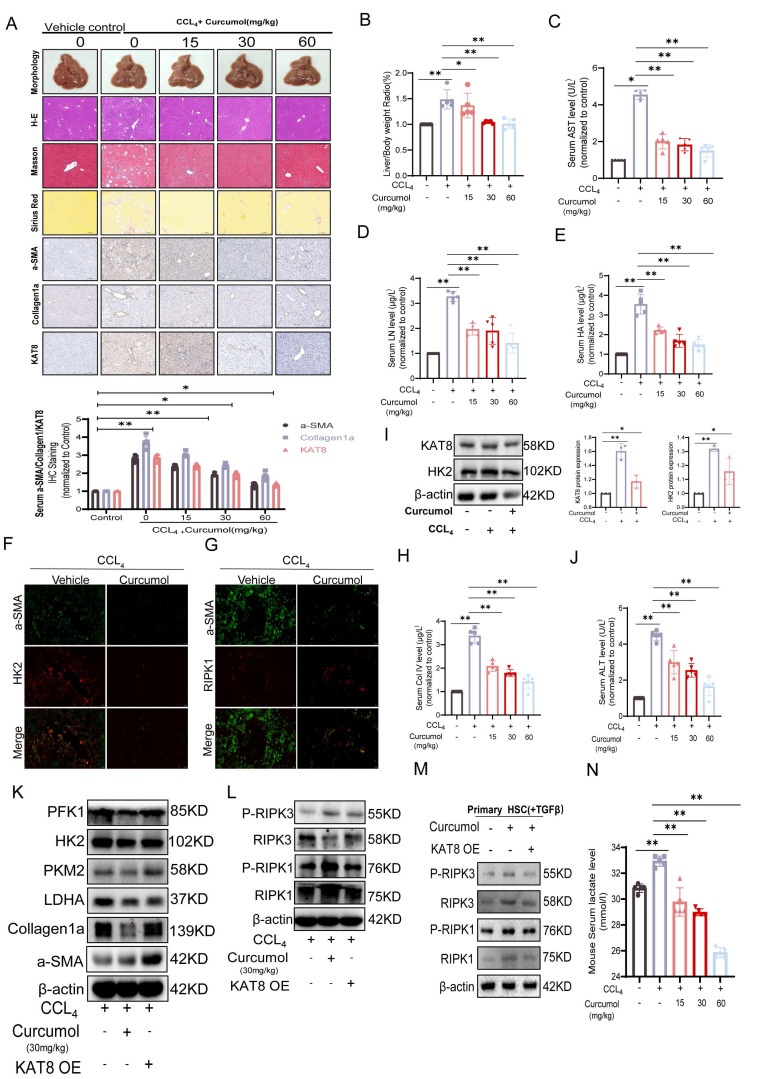
** Curcumol targets KAT8 to suppress glycolysis and induce necroptosis in hepatic stellate cells (HSCs) *in vivo*.** A. Representative images of liver sections from vehicle, different doses of Curcumol, and CCl₄-induced fibrotic mice (n = 5), stained with hematoxylin-eosin (H&E), Masson, and Sirius Red, and analyzed by immunohistochemistry (IHC) for α-SMA, Collagen I, and KAT8. Scale bar = 200 μm. B. Liver-to-body weight ratios of mice in the CCl₄ model group, Curcumol treatment group (n = 5). C-E. Serum levels of aspartate aminotransferase (AST), laminin (LN), and hyaluronic acid (HA) in CCl₄-induced fibrotic mice treated with vehicle, measured by biochemical assays (n = 5). F, G. Immunofluorescence analysis of colocalization between α-SMA (green) and HK2 (red), or α-SMA (green) and RIPK1 (red), in liver tissues from normal and CCl₄-induced fibrotic mice (n = 3). Scale bar = 20 μm. H,J. Serum levels of ALT and collagen IV (Col IV) in CCl₄-induced fibrotic mice treated with vehicle, Curcumol, measured by ELISA (n = 5). I. Western blot analysis of KAT8 and HK2 protein expression in liver tissues from vehicle-, Curcumol-, and CCl₄-treated mice, with densitometric quantification (n = 5). K-L. Western blot analysis of α-SMA, Collagen I, PFK1, PKM2, HK2, LDHA, RIPK1, RIPK3, phosphorylated RIPK1 (p-RIPK1), and phosphorylated RIPK3 (p-RIPK3) in liver tissues from vehicle-, Curcumol-, and KAT8 OE + Curcumol-treated CCl₄-induced fibrotic mice (n=3), with densitometric quantification. M. Western blot analysis of α-SMA, Collagen I, PFK1, PKM2, HK2, LDHA, RIPK1, RIPK3, phosphorylated RIPK1 (p-RIPK1), and phosphorylated RIPK3 (p-RIPK3) in liver tissues from vehicle-, Curcumol-, and KAT8 OE + Curcumol-treated TGF-β induced primary HSCs (n=3), with densitometric quantification. N. Serum lactate levels in CCl₄-induced fibrotic mice treated with different doses of Curcumol, measured by lactate assay kits (n = 5). Data are shown as mean ± SD, and statistical differences were analyzed by one-way ANOVA. ns indicates no significance; *p < 0.05, **p < 0.01, ***p < 0.001.

*In vitro* studies showed that Curcumol inhibits HSC activation by targeting KAT8. To confirm these effects *in vivo* and explore the underlying mechanism, we used a carbon tetrachloride (CCl₄)-induced mouse model of liver fibrosis. Histological and immunohistochemical analyses revealed that Curcumol substantially alleviated liver injury and fibrosis, with dose-dependent reductions in α-SMA, type I collagen, and KAT8 expression (Fig. 8A). The liver-to-body weight ratio (Fig. 8B), serum biochemical markers (AST, LN, HA; Fig.8C-E), and ELISA measurements (ALT, ALP, PCIII, ColIV; Fig. 8H, J; Supplementary Data S4I-J) all confirmed the hepatoprotective effects of Curcumol. Immunofluorescence analysis showed that CCl₄ treatment markedly increased the co-localization of α-SMA, HK2, and RIPK1, which was significantly reduced by Curcumol (Fig. 8F,8G). Western blot analysis confirmed that Curcumol downregulated the expression of α-SMA, type I collagen (Fig. 8K, Supplementary Data S4E) and key glycolytic proteins, including HK2, PFK1, PKM2, and LDHA (Fig. 8K, Supplementary Data S4F), and also reduced serum lactate levels in fibrotic mice (Fig. 8N). Furthermore, Curcumol upregulated the expression of RIPK1 and RIPK3, and their phosphorylated forms (Fig. 8L, Supplementary Data S4G). Similar results were observed in primary mouse HSCs (Fig. 8M, Supplementary Data S4H). In contrast, KAT8 overexpression fully reversed the effects of Curcumol *in vivo*. These findings reveal that Curcumol suppresses glycolysis in a KAT8-dependent manner while promoting necroptosis. Safety assessments in healthy mice showed no significant differences between Curcumol-treated and control groups in H&E, Masson, or Sirius Red staining. In addition, the liver-to-body weight ratio and serum biochemical parameters were unchanged, confirming that Curcumol exhibited no detectable toxicity at the experimental doses.

**Figure 9:**
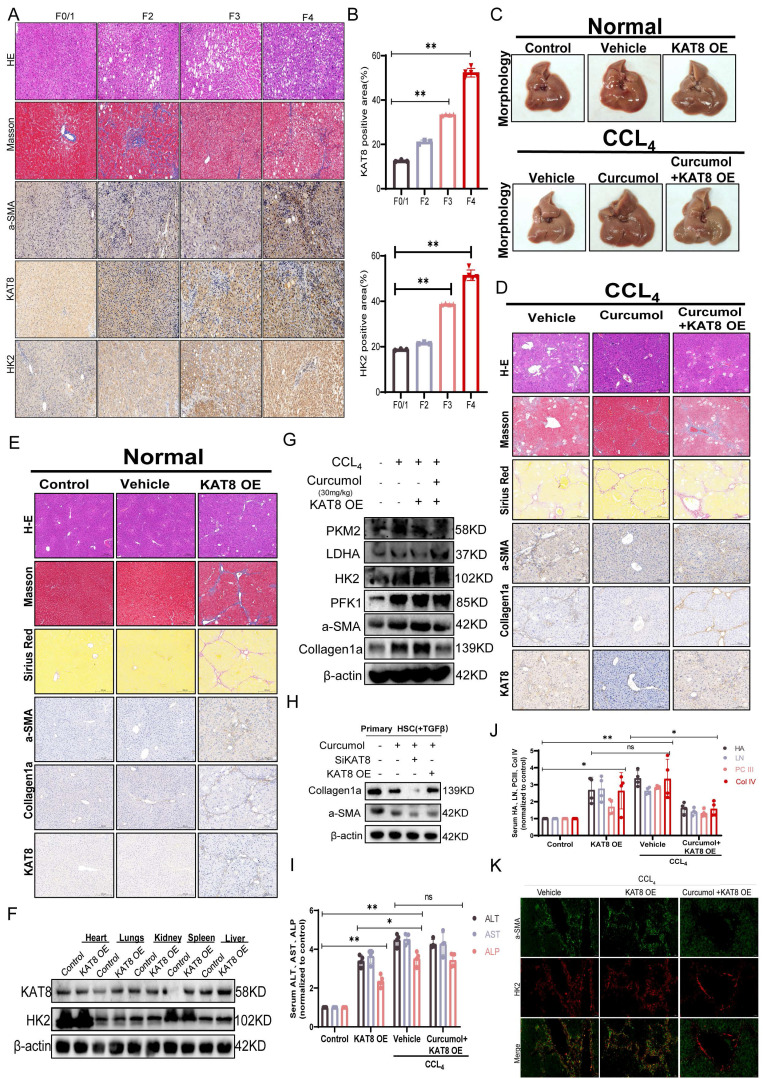
** KAT8 promotes hepatic fibrosis *in vivo* via HK2-mediated regulation of RIPK1-dependent necroptosis.** A. Liver fibrosis staging was performed using the Ishak score. For histopathological analysis, H&E, Masson, and IHC were used to stain a-SMA, HK2 and KAT8 in 15 human liver samples (F0/1, 3; F2, 3; F3, 4; F4, 5). Representative images are shown. Scale bar: 200µm, n=6/group. B. Quantification of positive KAT8 and HK2 IHC staining (n= 6 / group). C-E. Liver tissues from CCl4-induced fibrotic mice (n=5) treated with vehicle, Curcumol, or KAT8 overexpression combined with Curcumol (KAT8 OE + Curcumol 30mg/kg) were subjected to hematoxylin and eosin (H&E) staining, Masson's trichrome staining, Sirius Red staining, and immunohistochemical (IHC) analysis for α-smooth muscle actin (α-SMA), collagen I, and KAT8. Scale bar = 200 μm. F. Protein expression of KAT8 and hexokinase 2 (HK2) in heart, liver, lung, kidney, and spleen tissues from CCl4-induced fibrotic mice (vehicle and KAT8 OE groups, n=5) was quantified by immunoblotting (n=3). G. Western blot analysis was performed to determine protein levels of α-SMA, collagen I, pyruvate kinase M2 (PKM2), phosphofructokinase-1 (PFK1), HK2, and lactate dehydrogenase A (LDHA) in liver tissues from vehicle, Curcumol, KAT8 OE, and KAT8 OE + Curcumol (30mg/kg) groups (n=5). H. Primary mouse hepatic stellate cells (HSCs) were subjected to KAT8 knockdown or overexpression, and immunoblotting was performed to assess collagen I and α-SMA expression after 24 h of Curcumol (30mg/kg) treatment (n=3; scale bar: 10 μm). I. Serum biochemical parameters, including aspartate aminotransferase (AST), alanine aminotransferase (ALT)and alkaline phosphatase (ALP) were evaluated in vehicle, Curcumol (30mg/kg), KAT8 OE, and KAT8 OE + Curcumol (30mg/kg) groups (n=5). J. Enzyme-linked immunosorbent assay (ELISA) was used to measure serum levels of hyaluronic acid (HA), laminin (LN), procollagen III (PCIII), and collagen IV (Col IV) in the same groups (n=5). K. Immunofluorescence analysis was performed to examine the co-localization of α-SMA (green) and HK2 (red) in liver tissues from CCl4-induced fibrotic mice (vehicle, KAT8 OE, and KAT8 OE + Curcumol (30mg/kg) groups, n=3). Scale bar = 20 μm. Data are shown as mean ± SD, and statistical differences were analyzed by one-way ANOVA. ns indicates no significance; *p < 0.05, **p < 0.01, ***p < 0.001.

To further investigate the roles of KAT8 and HK2 in the pathological progression of liver fibrosis, we examined their expression in clinical human liver fibrosis tissue samples. Histological analysis by H&E staining, Masson staining, and immunohistochemistry for α-SMA (a marker for activated hepatic stellate cells) revealed typical fibrotic features in patient liver tissues (Fig. 9A). Subsequent semi-quantitative analysis based on the Metavir scoring system demonstrated a significant positive correlation between KAT8 and HK2 expression in fibrotic liver tissues (Fig. 9B). Mechanistic studies demonstrated that KAT8 overexpression (KAT8-OE) significantly exacerbated CCl₄-induced liver fibrosis, as shown by an increased liver-to-body weight ratio (Supplementary Data S5A); greater collagen deposition, assessed by H&E, Masson, and Sirius Red staining (Fig. 9C, 9E); and elevated expression of α-SMA and type I collagen, along with higher serum markers of fibrosis and liver injury (Fig. 9I-J). Conversely, Curcumol treatment alleviated these pathological changes (Fig. 9D), but KAT8-OE fully negated its protective effects, highlighting the pivotal role of KAT8 in fibrosis progression.

Further analysis showed that KAT8 OE increased hepatic HK2 protein levels (Fig. 9F, Supplementary Data S5C,5D), upregulated glycolytic enzymes (LDHA, PKM2, and PFK1) and fibrosis-related proteins (Fig. 9G, Supplementary Data S5E,5F), and these effects were confirmed in primary HSCs (Fig. 9H, Supplementary Data S5G). This was accompanied by a significant increase in serum lactate levels (Supplementary Data S5B). Immunofluorescence analysis further demonstrated enhanced co-localization of α-SMA and HK2 in fibrotic livers, which was reduced by Curcumol treatment but fully restored by KAT8 OE (Fig. 9K).

Overall, the *in vivo* and *in vitro* results indicate that Curcumol suppresses HSC glycolysis by targeting KAT8, alleviates HK2-mediated inhibition of necroptotic signaling, and activates the RIPK1/RIPK3 pathway to induce necroptosis, thereby producing strong anti-fibrotic effects.

## 3. Discussion

From both traditional Chinese and western medicine perspectives, HSC activation is the key mechanism underlying “stasis”^21^. Using blood-activating and stasis-resolving Chinese herbal medicines to inhibit HSC activation or induce HSC death offers a promising strategy for treating liver fibrosis^22^. A thorough review of existing literature combined with data analysis indicates that Curcuma zedoaria ranks third in usage frequency among blood-activating and stasis-resolving herbs^23^. This highlights its recognized importance as a key component in the treatment of chronic liver disease. Curcuma is the dried rhizome of three perennial herbaceous plants in the Zingiberaceae family: Curcuma zedoaria, Curcuma guangxiensis, and Curcuma longa^24^. Rhizomes are described as having a pungent, bitter, and warm taste and are traditionally associated with the meridians of the liver and spleen^25^. Curcuma is valued for its ability to resolve blood stasis, promote qi circulation, reduce masses, and relieve pain, and it is widely used to treat liver fibrosis and cirrhosis^26^. Recent studies have demonstrated that the active components of Curcuma are mainly volatile oils, including curcumone, Curcumol, and oleoresin^27^. Curcumol is highly effective in treating chronic liver disease because it can inhibit HSC migration and adhesion and suppress abnormal vascular proliferation in the liver^28^. These results highlight the significant potential of Curcumol as an anti-fibrotic agent and support the need for further research into its therapeutic mechanisms.

Lactic acid has long been considered merely the end product of glycolysis, typically removed from the body through excretion^29^. However, recent studies indicate that lactic acid has broader biological roles. Beyond serving as a substrate for energy metabolism and a modulator of immune responses^30^, lactic acid functions as a signaling molecule that regulates various non-histone proteins through lactylation^31^. This post-translational modification covalently attaches lactic acid to lysine residues, dynamically controlling the stability, activity, and subcellular localization of target proteins^32^. Lactic acid levels are significantly higher in fibrotic liver tissue than in normal tissue, providing a favorable environment for sustained HSC activation. Therefore, targeting and inhibiting lactic acid production has emerged as a potential therapeutic strategy for treating liver fibrosis^11^, ^26^, ^33^.

This study demonstrated that lactate produced through glycolysis in activated HSCs promotes KAT8-mediated lactoylation at the HK2 K346 site, establishing a “lactate-KAT8-HK2” positive feedback loop. This modification inhibits HUWE1-mediated ubiquitination and degradation of HK2, maintaining high HK2 expression and enhancing glycolytic activity. For the first time, protein lactylation is implicated in liver fibrosis, revealing a novel mechanism in which the metabolic intermediate lactate regulates enzyme function through epigenetic modification. The HK2 K346 site is highly conserved evolutionarily, and its mutation results in loss of enzymatic activity. Importantly, this mutation completely prevents Curcumol-induced necroptosis in HSCs, highlighting the essential role of K346 as a metabolic checkpoint.

Curcumol exerts targeted regulation of the metabolic-death axis through a dual mechanism. First, it directly inhibits the catalytic activity of KAT8, reducing HK2 lactylation and triggering proteasome-dependent degradation of key glycolytic enzymes (HK2, PKM2, and LDHA), which disrupts the energy supply in HSCs. Second, by decreasing HK2 levels, it alleviates HK2-mediated inhibition of RIPK1 ubiquitination, thereby activating the RIPK1/RIPK3 necroptosis signaling pathway. This combined “metabolic deprivation-cell death” effect effectively overcomes the compensatory rebound that is often observed with single-pathway interventions. *In vitro* and *in vivo* studies have shown that this approach selectively targets and eliminates activated HSCs without significant toxicity to normal hepatocytes, indicating a high degree of therapeutic precision and safety. Notably, we also observed that curcumol downregulates the mRNA levels of HK2 and other key enzymes in the glycolytic pathway. This suggests the possible existence of a multi-layered regulatory network, and the specific transcriptional mechanisms involved warrant further investigation in future studies.

KAT8 is highly expressed in fibrotic liver tissue and colocalizes with HK2 and RIPK1, confirming its activation within the pathological microenvironment. KAT8 overexpression abolishes the antifibrotic effects of Curcumol, whereas KAT8 inhibitors reproduce the Curcumol phenotype, highlighting the core driving role of the KAT8-HK2 axis. From a translational perspective, KAT8, a member of the histone acetyltransferase family, is a promising target for small-molecule inhibitor development. This study further identified that Curcumol binds to the TYR-361 and THR-319 sites of KAT8 via molecular docking, thereby providing a theoretical foundation for future structural optimization.

Previous studies have shown a relationship between HSC activation and enhanced Warburg effect^34^. However, most metabolic interventions have focused on inhibiting individual glycolytic enzymes^35^, rather than addressing the overall metabolic reprogramming associated with the Warburg effect^36^. The key findings of this study are as follows: first, lactate acts as a metabolic signaling molecule, controlling HK2 stability via KAT8-mediated protein lactylation; second, HK2 functions not only as a rate-limiting glycolytic enzyme but also as a regulator of apoptotic signals through its mitochondrial binding, with its loss triggering necroptosis in HSCs; and third, Curcumol selectively eliminates HSCs through coordinated regulation across metabolism, epigenetics and cell death pathways. These results closely align with the concept of “metabolic checkpoint regulation of cell fate” and introduce a novel therapeutic framework for liver fibrosis.

Curcumol, the active component of the traditional Chinese medicine Curcuma, exhibits well-characterized pharmacokinetics and adjustable bioavailability^37^. Our *in vivo* and *in vitro* experiments indicate that Curcumol exhibits favorable tissue-targeting properties. In the CCl4-induced liver fibrosis model, Curcumol concentrations were significantly higher in fibrotic liver tissues than in normal liver tissue, which may explain its selective action on activated HSCs. As a small-molecule compound, Curcumol also shows suitable pharmacokinetic features, including an appropriate half-life and bioavailability, supporting its potential for clinical application. Furthermore, this study confirmed that Curcumol significantly improves liver fibrosis markers in the CCl4-induced mouse model without causing detectable toxicity to normal liver tissue. This finding provides important safety evidence for clinical translation.

## 4. Materials and Methods

### Patient specimens

This study analyzed liver tissue samples collected from patients treated at Nanjing Hospital of Traditional Chinese Medicine, affiliated with Nanjing University of Traditional Chinese Medicine, between February 2013 and June 2017. Written informed consent was obtained from all participants, and the study protocol was approved by the Institutional Review Board of the hospital. All procedures were conducted in accordance with the Declaration of Helsinki. Histopathological assessments were performed on paraffin-embedded tissue sections, including immunohistochemical staining for α-SMA, HK2, and KAT8. The fibrosis stages were classified according to the Metavir scoring system: no fibrosis (F0/1, n = 3), moderate fibrosis (F2, n = 3), and advanced fibrosis (F3/F4, n = 9). Detailed patient demographic and clinical data have been previously reported^38^.

### Animal experiments

This study was conducted at the Animal Experiment Center of Nanjing University of Chinese Medicine and received approval from the University's Animal Ethics Committee (approval number: 202412A065). Eight-week-old SPF-grade male ICR mice weighing approximately 20 g were obtained from Hangzhou Medical College. Mice were housed in a controlled airflow cabinet and provided with ad libitum access to food and water. The rAAV-GFAP-KAT8-P2A-EGFP-WRES viral vector (5.00 × 10¹² vg/ml), supplied by Brain VTA, was used to overexpress KAT8 in the liver. The viral vector was delivered through tail vein injection, simultaneously with intraperitoneal administration of CCl₄ to induce liver fibrosis. CCl₄ (Sigma, St. Louis, MO, USA) was prepared as a 10% solution and injected intraperitoneally at a dose of 0.5 μL/g body weight, three times per week for eight consecutive weeks.

Curcumol was first dissolved in polyethylene glycol 300 (PEG300) and then gradually combined with vegetable oil. A 5% Tween 80 solution was added as an emulsifier, and the mixture was subjected to ultrasonic treatment or high-speed stirring to produce a stable emulsion. The final solvent comprised 50% vegetable oil, 45% PEG300, and 5% Tween 80.

After one week of acclimatization, the mice were randomly assigned to two main experimental cohorts. Normal control group: subdivided into normal control, normal control + empty plasmid, normal control + KAT8 overexpression, and normal control + middle-dose Curcumol. CCl₄-induced liver fibrosis model group: subdivided into CCl₄ + empty plasmid, CCl₄ + low-dose Curcumol, CCl₄ + middle-dose Curcumol, CCl₄ + high-dose curcumol, CCl₄ + KAT8 overexpression, CCl₄ + high-dose Curcumol + empty plasmid, and CCl₄ + high-dose Curcumol + KAT8 overexpression.

After model establishment, mice received low-dose Curcumol (15 mg/kg) or middle-dose Curcumol (30 mg/kg), with CCl₄ administered every other day throughout the treatment period. Each experimental group included six ICR mice. Animals were euthanized following approved ethical guidelines, and tissue samples were collected for subsequent analysis.

### Cell culture

The following cell lines were obtained from the Cell Bank of the Chinese Academy of Sciences (Shanghai, China): LX-2 (human hepatic stellate cell line, RRID: CVCL_5792), THLE-2 (Human liver epithelial cell line, RRID: CVCL_3803), AML12 (mouse hepatocyte cell line, RRID: CVCL_0140), RAW264.7 (mouse macrophage cells, RRID: CVCL_0493), THP-1 (human acute monocytic leukemia cells, RRID: CVCL_0006), LSEC (liver sinusoidal endothelial cells, RRID: CVCL_QY34), and HEK-293T (human embryonic kidney 293T cells, RRID: CVCL_0063). Cells were purchased on the following dates: December 2023 (LX-2, LSEC), March 2024 (THLE-2, THP-1, RAW264.7), and June 2023 (AML12, HEK-293T). All cell lines were authenticated by short tandem repeat profiling at the time of purchase, and mycoplasma contamination was routinely screened using PCR-based methods, with all tests returning negative results. Only authenticated, mycoplasma-free cells were used in the experiments. Cells were cultured according to the supplier's instructions, and all experiments were conducted within 10 passages after thawing.

LX2 cells (obtained from the Shanghai Cell Bank of the Chinese Academy of Sciences) were cultured in incomplete Dulbecco's Modified Eagle Medium (DMEM), whereas Thle-2 cells were cultured in incomplete DMEM/F12 medium supplemented with 10% fetal bovine serum (FBS) and 1% penicillin-streptomycin. During the experiment, the cell lines used included THLE-2, LX2, AML12, THP-1, RAW264.7, LSEC, and HEK-293T. FBS was purchased from Kirgen Bioscience (Shanghai), and DMEM was sourced from NEST Biotechnology.

Primary HSCs were isolated from adult ICR mice using a modified protocol adapted from previous studies. Four to five mice were selected, and retrograde hepatic perfusion was performed using a solution containing streptokinase (P5147, Sigma-Aldrich), collagenase (LS004194, Worthington), and deoxyribonuclease (LS002139, Worthington). After perfusion, livers were excised and mechanically dissociated to generate a cell suspension, which was filtered through a 70 μm cell strainer and collected into a 50 mL centrifuge tube. The suspension was centrifuged at 30×g for 5 min, and non-parenchymal cells were recovered from the supernatant. These cells were then centrifuged at 1000×g for 5 min, the supernatant discarded, and the pellet washed twice with Gey's balanced salt solution (GBSS)/B buffer^39^. HSCs were enriched via density gradient centrifugation using Nycodenz (AN1002424, Accurate Chemical and Scientific Corporation) at 1,380×g for 20 min without a brake. The purity of the isolated HSCs was confirmed by spontaneous retinol autofluorescence under a Zeiss LSM 700 confocal microscope^40^.

Kupffer cells were isolated from healthy and fibrotic model mice (34-45 g) using a standardized protocol^41^. After *in situ* perfusion with EGTA/HBSS and collagenase solutions, the livers were excised, incubated in Kupffer Cell Isolation Medium on ice for 3h, and gently agitated to release cells. The cell suspension was filtered and subjected to a series of centrifugations to remove hepatocytes, and the resulting supernatant was centrifuged to pellet the non-parenchymal cells. Kupffer cells were finally purified from the non-parenchymal cell fraction using a 25%/50% SIP density gradient, collected from the interface, washed, and seeded.

### CCK8 cell viability assay

Cells were seeded at a density of 3 × 10³ cells per well in a 96-well plate. After 24 h of drug treatment, cell viability and proliferation were assessed using the CCK8 assay kit. For the assay, 10 μL of CCK8 working solution was added to each well, followed by incubation at 37 °C in a 5% CO₂ incubator for 1 h. Subsequently, the optical density at 450 nm (OD₄₅₀) was measured using a microplate reader, and cell viability for each treatment concentration was calculated. The CCK8 assay kit (ZYCD002-0100) was obtained from ZUNYAN, Nanjing, China.

### Real-time quantitative polymerase chain reaction (RT-qPCR)

Total RNA was isolated from LX2 cells and tissue samples using Trizol reagent. RNA concentration was measured using a NanoDrop 2000 spectrophotometer. The purified RNA was reverse transcribed into complementary DNA (cDNA) and subsequently diluted tenfold for downstream applications. Quantitative PCR (qPCR) was conducted on a QuantStudio 6 Flex real-time fluorescent quantitative PCR instrument (Thermo Fisher Scientific, USA) using ABScript III RT pre-mix with a genomic DNA removal agent (Accurata Biotechnology, Changsha, China). The qPCR reaction program was as follows: Pre-denaturation at 95 °C for 3 min, followed by 40 cycles of denaturation at 95 °C for 5 s and annealing/extension at 60 °C for 30 s. The results were analyzed using the 2^(-ΔΔCT) method. Table 1 presents the corresponding primer sequences.

### Reagents

CQ (HY-17589A), Ferrostatin-1 (HY-100579), MG132 (HY-13259), TTM (HY-128530), Lox (HY-P2976), MG149 (HY-15887), and CHX(HY-12320) were purchased from MedChemExpress. Liproxstatin-1 (S7699) was purchased from Selleck.Z-VAD-FMK (A1902) were purchased from ApexBio.

### Protein western blotting

After 24 h of drug treatment, the culture supernatant was removed, and cells were washed twice with phosphate-buffered saline (PBS) at room temperature. Subsequently, cells were lysed on ice for 30 min using lysis buffer supplemented with phenylmethylsulfonyl fluoride (PMSF) and a phosphatase inhibitor to extract total protein. Protein concentrations were determined using the BCA assay, and 5× SDS-PAGE loading buffer was added to the samples for denaturation. Proteins were separated via SDS-PAGE gel electrophoresis (90 V for 20 min; then 120 V for 120 min) and transferred onto a 0.2 μm PVDF membrane at 110 V for 120 min. The membrane was blocked with blocking solution at room temperature for 2 h, then cut into sections and incubated with the appropriate primary antibodies. After washing, membranes were incubated with rabbit or mouse secondary antibodies (at a ratio of 1:10,000) at room temperature for two hours. The protein bands were detected using enhanced chemiluminescence (ECL) and analyzed using a chemiluminescence gel imaging system.

The antibodies employed in this study include: RIPK1 (17519-1-AP), RIPK3 (17563-1-AP), P-RIPK1 (66854-1-Ig), MLKL (21066-1-AP), P-MLKL (82090-2-RR), HK2 (22029-1-AP), PKM2 (15822-1-AP), LDHA (19987-1-AP), PFKM (55028-1-AP), HUWE1 (19430-1-AP), Ubiquitin (10201-2-AP), HA (51064-2-AP), His (66005-1-Ig), EP300 (20695-1-AP) and P62 (18420-1-AP) from proteintech; KAT8 (sc-271691) from Santa Cruz Biotechnology; KAT6A from Bioswamp; Collagen I (ab26003), β-actin (ab8226), Tubulin (Ab721), anti-mouse IgG (ab190475), anti-rabbit IgG (ab288151) and LC3B (ab192890) from Abcam; Pan-Kla (AB_2868521) from PTM-Bio laboratory; KAT6B(A17116), P-RIPK3(AP1260), AARS(A15017), and AARS2 (A7826) from Abclonal. Chemical reagents included ECL reagent (BMU102) from Abbkine, TBST from Swiss Affinibody LifeScience AG, 2-Deoxy-D-glucose (2-DG), MG-132 (M8699), CCl₄ (SRP3115), and chloramphenicol (CHX, AB9014) from Selleck Chemicals; Acetylated-Lysine antibody (AB_331805) from Cell Signaling Technology; and recombinant TGFβ1 (H8541) and sodium lactate from Sigma-Aldrich.

### Immunoprecipitation (Co-IP) and mass spectrometry (MS)

Cells were first rinsed with ice-cold PBS and then lysed using an immunoprecipitation (IP) buffer supplemented with glycerol phosphate, 1.5 mM MgCl2, and 2 mM ethylenediaminetetraacetic acid (EGTA). The lysates were cleared by centrifugation at 12,000 × g for 20 min at 4 °C to remove cell debris and unlysed materials. Antibody-bound protein complexes were enriched by incubating the lysates with either control IgG or the specific primary antibody in IP buffer supplemented with protease inhibitors at 4 °C for 24 h. Subsequently, 30 μL of Protein G beads (Roche) were added, and the mixture was incubated at 4 °C for an additional 12 h. After incubation, the samples were centrifuged at 2,000 × g for 5 min to remove the supernatant. The magnetic beads were washed four times with IP buffer to eliminate non-specific protein interactions. Bound proteins were denatured by heating the beads at 95°C for 10 min, followed by SDS-PAGE separation and staining with Coomassie Brilliant Blue R-250 (Bio-Rad). The gel bands corresponding to the target protein were excised, destained, and digested with trypsin. The resulting peptides were analyzed and identified using liquid chromatography-tandem mass spectrometry (LC-MS/MS) on an Orbitrap Velos Pro mass spectrometer (Thermo Fisher Scientific).

### Drug affinity responsive target stability (DARTS)

Total protein was extracted from LX-2 human hepatic stellate cells using RIPA lysis buffer supplemented with a protease inhibitor cocktail, and the concentration was determined by BCA assay. Equal amounts of total protein were incubated with varying concentrations of Curcumol (dissolved in DMSO) or an equal volume of DMSO vehicle control at room temperature for 1 hour. Subsequently, each sample was digested with an optimized concentration of Pronase (Catalog # P5147, Sigma-Aldrich) at room temperature for 30 minutes. The digestion reaction was terminated by adding pre-cooled EDTA to a final concentration of 5 mM. Finally, the stability of KAT8 protein was assessed by Western blot analysis.

### SiRNA, overexpression plasmid, and site-directed mutagenesis plasmid transfection

LX2 cells were seeded into a 6-well plate and allowed to reach 40%-50% confluence before initiating transfection. Transfection was performed using either SuperKine Lipo3.0 transfection reagent (Abbkine) or LNP30i Mate (LTR0301, BiOligo Biotechnology, Shanghai), in combination with optimized minimal essential medium (Opti-MEM), in accordance with the manufacturer's instructions. siRNA was diluted in Opti-MEM and mixed with the transfection reagent. The mixture was then incubated at room temperature for 10-15 min to allow formation of siRNA-lipid complexes. Subsequently, these complexes were added to the cells. After 36 h, the medium was replaced with fresh culture medium, and cells were collected after 72 h for subsequent experiments.

The siRNA sequences used in this study are listed as follows: KAT6A (CGGCGCTATACTAATCCAATA), KAT6B (CCCAAACGTATGCGTCGTAAA), EP300 (CAATTCCGAGACATCTTGAGA), and KAT8 (CGAAATTGATGCCTGGTATTT). Additional genes targeted included AARS2 (CCATCATACCTTCTTTGAAAT), KAT2A (GCTGAACTTTGTGCAGTACAA), HUWE1 (TTGGACCGCTTCGATGGAATA), and RIPK1 (GGGAAGGTGTCTCTGTGTTTC). The expression plasmids employed in this study comprised pEnCMV-HK2 (human)-3×HA, pENTR1A-Huwe1, and pEnCMV-KAT8 (human)-3×His.

### Transmission electron microscopy characterization

Samples were examined using a JEOL JEM-2100 transmission electron microscope (bright field imaging mode, 200 kV acceleration voltage). Samples were cut into approximately 80-nm-thick sections using an ultramicrotome and subsequently stained with uranyl acetate and lead citrate to improve contrast. Images were captured with a Gatan digital camera and processed and analyzed using DigitalMicrograph software.

### Non-targeted metabolomics analysis

In accordance with the protocol described in Reference^42^, a non-targeted metabolomics analysis was performed to assess metabolite changes in HSCs. HSCs were grown in large culture dishes to confluence, treated with the drug, and incubated for 24 h. After treatment, the cells were thoroughly washed with PBS, digested with trypsin, and centrifuged at 1,000 rpm for 3 min to remove the supernatant. The cells were resuspended in PBS, and this centrifugation and washing process was repeated three times. Finally, the cell pellets were stored at -80 °C and sent to a professional biotechnology company for metabolomics analysis.

### Immunofluorescence staining

HSCs were seeded into a 24-well culture plate and fixed with 4% paraformaldehyde for 15 mins. The cells were then permeabilized with 0.1% Triton X-100. Subsequently, the appropriate primary antibody was applied, and the mixture was incubated at 4 °C overnight. The following day, cells were incubated with a FITC-conjugated secondary antibody at 37 °C for 2 h. The cell nuclei were counterstained with DAPI (KeyGEN BioTECH, KGA215-50), and fluorescence images were captured using a fluorescence microscope.

### Immunohistochemistry

Fresh tissue specimens were fixed in 4% paraformaldehyde, dehydrated using a graded ethanol series, cleared with xylene, and embedded in paraffin. After dewaxing, the sections were subjected to antigen retrieval and nonspecific site blocking. Subsequently, primary and secondary antibodies were applied, followed by DAB staining, and nuclei were counterstained with hematoxylin. Images were captured using a microscope, and DAB signals were quantified in ImageJ software by setting an appropriate threshold. Subsequently, the percentage of positive staining was calculated. Hematoxylin staining was used to facilitate the enumeration of total cell nuclei, thereby ensuring a comprehensive assessment of the entire tissue area. Mean values from multiple fields of view were used to generate representative quantitative data for statistical analysis.

### Immunoprecipitation

After 24 h of drug treatment, cells were washed twice with PBS at room temperature and lysed on ice for 30 min using lysis buffer (containing PMSF and phosphatase inhibitors) to extract total protein. The lysate was pre-cleared with protein A/G agarose or magnetic beads, then incubated with a primary antibody specific to the target protein to allow the formation of antigen-antibody complexes. Protein A/G magnetic beads were added to capture these complexes, which were collected via centrifugation or magnetic separation. Following multiple washes to remove non-specific binding proteins, the complexes were eluted by boiling or with an elution buffer. Finally, the immunoprecipitation proteins were resolved by SDS-PAGE and analyzed by western blot.

### CETSA assay

Cellular Thermal Shift Analysis (CETSA) is an approach employed to evaluate the binding affinity of small-molecule compounds to intracellular target proteins. The principle of this technique is that when the target protein binds to the ligand, its thermal stability increases, causing a rightward shift of the thermal desorption curve. In this experiment, LX2 cells were treated with or without Curcumol for 3 h and then divided into eight equal portions. Each portion was exposed to different temperatures (43 °C, 46 °C, 49 °C, 52 °C, 55 °C, 58 °C, 61 °C, and 64 °C) for 3 min. Subsequently, the samples were stored at -80 °C for 12 h, thawed at room temperature for 5 min, and subjected to two additional freeze-thaw cycles to achieve complete lysis. After centrifugation at 16,000 × g for 20 min, the supernatant was collected for protein stability assessment. KAT8 protein levels were then measured by western blot, and thermolysis curves were generated for comparative analysis.

### Protein affinity purification

To express the recombinant protein, we transformed plasmids encoding a His-tagged KAT8 fusion protein into E. coliBL21(DE3) cells. The transformed bacteria were grown in Luria-Bertani (LB) medium containing the appropriate antibiotics at 37 °C until the OD600 reached 0.6-0.8. Protein expression was induced by adding 0.5 mM isopropyl β-D-thiogalactopyranoside (IPTG), after which the culture was incubated overnight at 16 °C to promote proper protein folding and stability. The cells were subsequently harvested and lysed by ultrasonication in a lysis buffer composed of 50 mM HEPES (pH 7.4), 150 mM NaCl, and 1 mM PMSF. Following high-speed centrifugation to remove insoluble debris, the supernatant was loaded onto a Ni-NTA affinity column for purification. Nonspecifically bound proteins were washed off using a low-concentration imidazole buffer, and the target His-KAT8 protein was eluted with elution buffer containing 250 mM imidazole. The concentration of the eluted protein was estimated based on fluorescence intensity, and its purity was evaluated by SDS-PAGE analysis.

### H&E staining

Tissue samples were initially fixed in 4% paraformaldehyde for 48 h, followed by dehydration in 70% ethanol for 12 h. The samples were then trimmed into approximately 0.5 cm^3^ blocks, further dehydrated, and embedded in paraffin. Paraffin-embedded blocks were sectioned, baked, and dewaxed before undergoing hematoxylin and eosin (H&E) staining. After drying and mounting, six randomly selected fields per section were observed and photographed under a light microscope for histological evaluation.

### Proteomics analysis and functional annotation

Total cellular proteins were extracted using RIPA lysis buffer, and protein concentrations were measured via the BCA assay. Equal amounts of protein were separated by SDS-PAGE and stained with Coomassie Brilliant Blue R-250. Target protein bands were excised, destained, reduced, alkylated, and digested with trypsin. The resulting peptides were separated by high-performance liquid chromatography (HPLC) and analyzed using liquid chromatography-tandem mass spectrometry (LC-MS/MS) on an Orbitrap Velos Pro mass spectrometer (Thermo Fisher Scientific). Mass spectrometry data were processed with MaxQuant software and matched against species-specific protein sequences from the UniProt database. Differential protein expression was assessed using tandem mass tag labeling or unlabeled quantification approaches. Subsequently, bioinformatic analyses, including GO and KEGG pathway enrichment, were performed to elucidate the roles of differentially expressed proteins in biological processes, molecular functions, cellular components, and signaling pathways.

### Extracellular acidification rate (ECAR) assay

The ECAR of cells was measured using the Agilent Seahorse XF Glycolytic Stress Assay Kit (103020-100, Seahorse Biosciences). The procedure was as follows: A total of 6 × 10⁴ cells were seeded into XFe96 microplates (101085-004, Seahorse Bioscience) and cultured at 37 °C with 5% CO₂ for 12 h. Before measurement, the cells were washed with XF Basement Medium (102353-100, minimal DMEM) and incubated in a CO₂-free environment at 37 °C for 1 h. During the ECAR assay, glucose (10 mM), oligomycin (1 μM), and 2-deoxyglucose (2-DG, 50 mM) were sequentially injected at defined time points. All experiments were performed in triplicate, and the ECAR values were normalized to total protein content. Data analysis, including assessment of basal glycolysis, glycolytic capacity, and glycolytic reserve, was performed using Wave software (v.2.3.0, Seahorse Bioscience, Agilent Technologies) in accordance with the manufacturer's instructions.

### Histone extraction

Histones were extracted using a conventional acid extraction protocol as follows: First, cell pellets were resuspended in a hypotonic lysis buffer (10 mM Tris-HCl, pH 8.0; 1 mM KCl; 1.5 mM MgCl2; 1 mM DTT) and incubated at 4 °C for 30 min. After centrifugation to isolate cell nuclei, the pellets were resuspended in 0.4 N H_2_SO_4_ and incubated at 4 °C for approximately 4 h. Subsequently, 1 volume of 100% trichloroacetic acid was added to twice the sample volume, yielding a final concentration of 33%. The mixture was then precipitated on ice for 1 h. The precipitate was washed twice with ice-cold acetone, air-dried, and the protein concentration was quantified at 230 nm. Equal amounts of histone proteins were loaded onto a 15% SDS-PAGE gel for electrophoresis, and total histone H3 was detected via western blotting to confirm uniform loading across all samples.

### Flow cytometry

LX2 cells were exposed to Curcumol at concentrations of 20, 30, and 45 μM for 24 h. After treatment, mitochondrial membrane potential was evaluated using a mitochondrial membrane potential assay kit (Beyotime, C2001S). Subsequently, the cells were analyzed by flow cytometry, and the data were processed with FlowJo software to precisely quantify alterations in mitochondrial membrane potential.

### HK2 activity assay

Mitochondria were isolated following the manufacturer's protocol using a mitochondrial extraction kit (SM0020, Solarbio). First, 5 × 10⁷ LX2 cells were collected and resuspended in 1 mL of lysis buffer. Subsequently, the resuspended cells were transferred to a pre-chilled Dounce homogenizer and homogenized 40 times. The homogenate was centrifuged at 1,000 × g for 5 min at 4°C, and the supernatant was collected and further centrifuged at 12,000 × g for 10 min. The precipitated mitochondria were washed twice with washing buffer and resuspended in storage buffer. HK activity was then measured using a hexokinase colorimetric assay kit (K789-100, Biovision).

### SPR affinity assay

The binding affinity and kinetic parameters between KAT8 protein and Curcumol were determined using surface plasmon resonance (SPR) technology. In the assay, KAT8 was immobilized onto the surface of an SPR sensor chip, and a series of Curcumol solutions at concentrations of 48.83 nM, 390.62 nM, 6250 nM, 12500 nM, 50000 nM, and 100000 nM were injected over the chip surface. Binding of Curcumol to the immobilized protein resulted in changes in the refractive index at the sensor surface, which were detected as real-time SPR response signals. The response signals, expressed in resonance units (RU), were recorded over time to generate sensorgrams. Binding kinetics were analyzed using a multicycle approach, in which association and dissociation phases were monitored for each concentration. The resulting data were fitted to a Langmuir binding model or other appropriate interaction models to determine the association rate constant (kₐ or kₒₙ), dissociation rate constant (kₑ or kₒff), and equilibrium dissociation constant. Finally, the binding strength and specificity of Curcumol toward KAT8 were evaluated through comprehensive analysis of the kinetic curves and fitted parameters.

### LDH activity measurement

LDH activity in HSCs was measured using a commercial colorimetric assay kit (Abcam, Cat. No. ab102526). After PBS washes, cells were lysed in the provided buffer, and the lysates were clarified by centrifugation at 12,000 × g for 10 min at 4 °C. A 50 µL aliquot of the supernatant was mixed with an equal volume of the reaction mixture and incubated at room temperature for 30 min under light-protected conditions. Optical density was measured at 450 nm using a microplate spectrophotometer, and the LDH activity was calculated based on an NADH standard curve, expressed in mU/mL.

### Lactic acid assay

Lactic acid levels were measured using the Lactic Acid Colorimetric/Fluorescent Assay Kit (K6027-100, Biovision). Cells were seeded at a density of 1 × 10^5^ cells/well in a 12-well plate. After collecting the culture medium, the assay was conducted according to the manufacturer's instructions.

### ATP content assay

ATP levels were assessed *in situ* using the ATP Detection Kit (S0026, Beyotime). Cells were seeded at a density of 1 × 10^6^ cells/well in a 6-well plate, collected, and analyzed following the manufacturer's instructions.

### Glucose level measurement

Intracellular glucose levels were determined using the GOD/POD colorimetric glucose assay kit (S0202M, Beyotime). Cells were seeded at a density of 1 × 10⁶ cells/well in a 6-well plate, collected, and analyzed according to the manufacturer's protocol.

### Statistical analysis

All experiments were conducted in triplicate, with each comprising a minimum of three technical replicates. Data are expressed as the mean ± standard deviation (SD), and the exact sample sizes are provided in the respective figure legends. Before the statistical analysis, data normality was assessed using the Shapiro-Wilk test. For datasets following a normal distribution, comparisons among multiple groups were performed using one-way analysis of variance (ANOVA) followed by Tukey's post hoc test. For non-normally distributed data or datasets with sample sizes, nonparametric tests such as the Kruskal-Wallis H test were applied. Statistical analyses were performed using SPSS software (v17.0), and significance levels were defined as: ns (not significant), *p < 0.05, **p < 0*.01*, and ***p < 0.001.

## Supplementary Material

Supplementary figures.

## Figures and Tables

**Table 1:** Primer sequences

Primer Name	Primer sequence (5' to 3')	Base number
H-HAT1-F	AAGCCATTCGGAACCTTACTTC	21
H-HAT1-R	AGTGCCATCTTTCATCATCCAC	20
H-KAT2A-F	AGGAAAACCTGTGGTTGAAGG	22
H-KAT2A-R	CAGTCTTCGTTGAGATGGTGC	23
H-KAT2B-F	CTCTGCCTTAACTACTGGAAGC	21
H-KAT2B-R	GCCATCTGGTGTAATTGACCTTG	21
H-CREBBP-F	AACTGGCGCTATCTCATTCTGG	22
H-CREBBP-R	CTGTTCTGCAAGGGAGTTCCT	21
H-EP300-F	TTCCCCTAACCTCAATATGGGAG	23
H-EP300-R	GCCTGTGTCATTGGGCTTTTG	21
H-TAF1-F	GAAGCACCGTGAGCTTATACAG	22
H-TAF1-R	CACTCTTGGCTTGGTATCCATT	22
H-KAT5-F	GGGGAGATAATCGAGGGCTG	20
H-KAT5-R	TCCAGACGTTTGTTGAAGTCAAT	23
H-KAT6A-F	TGAGTGGATTTTGGAGGCCAT	21
H-KAT6A-R	GCTATTCGCCCAGGATTATCAG	22
H-KAT6B-F	GCCTTGCCTCCTATAAGGACC	21
H-KAT6B-R	TCCACATTGCGGAGATCATTAC	22
H-KAT7-F	ATTCTGGACTGAGCAAAGAACAG	23
H-KAT7-R	GTCATACTCGCTTGTCAGGTTTT	23
H-KAT8-F	GCTGGACGAGTGGGTAGAC	19
H-KAT8-R	TTTGGTTGCGAGTGATCTTGC	21
H-ELP3-F	ACGAGGCAGTCAAGTATTCTGA	22
H-ELP3-R	GCAGTAATCTGGTCTGGTTTCA	22
H-GTF3C4-F	ATGGGTTGCGATGCTAATGG	20
H-GTF3C4-R	AACATGAAAGTCTGACTGACCG	22
H-NCOA3-F	AGTGGACTAGGCGAAAGCTCT	21
H- NCOA3-R	GTTGTCGATGTCGCTGAGATTT	22
H-AARS-F	GGGGCAAACATAATGACCTGG	21
H-AARS-R	GCCAAACTCTTGGGTGAGGA	20
H-RIPK1-F	GGGAAGGTGTCTCTGTGTTTC	21
H-RIPK1-R	CCTCGTTGTGCTCAATGCAG	20
H-RIPK3-F	ATGTCGTGCGTCAAGTTATGG	21
H-RIPK3-R	CGTAGCCCCACTTCCTATGTTG	22
H-PFKM-F	AGCTGCCTACAACCTGGTGA	20
H-PFKM-R	TCCACTCAGAACGGAAGGTGT	21
H-a-SMA-F	CCGACCGAATGCAGAAGG	18
H-a-SMA-R	ACAGAGTATTTGCGCTCCGGA	21
H-Collagen I-F	CCTCAAGGGCTCCAACGAG	19
H-Collagen I-R	TCAATCACTGTCTTGCCCCA	20
H-PKM2-F	TACCATGCGGAGACCATCAA	20
H-PKM2-R	AGCAACGGGCCGGTAGAG	18
H-PFK1-F	GGGGATGCTCAAGGTATGAAC	21
H-PFK1-R	TCGGCCTCTGCGATGTTTG	19
H-LDHA-F	TTGGTCCAGCGTAACGTGAAC	21
H-LDHA-R	CCAGGATGTGTAGCCTTTGAG	21
H-GAPDH-F	TGACATCAAGAAGGTGGTGAAGCAG	25
H-GAPDH-R	GTGTCGCTGTTGAAGTCAGAGGAG	24
H-HK2-F	GATTGCCTCGCATCTGCTTG	20
H-HK2-R	GCTCCAAGCCCTTTCTCCAT	20

## References

[1] Afonso MB, Rodrigues PM, Simão AL, Gaspar MM, Carvalho T (2016). Activation of necroptosis in human and experimental cholestasis. Cell Death Dis.

[2] Marcellin P, Kutala BK (2018). Liver diseases: A major, neglected global public health problem requiring urgent actions and large-scale screening. Liver Int.

[3] Henderson NC, Rieder F, Wynn TA (2020). Fibrosis: from mechanisms to medicines. Nature.

[4] Higashi T, Friedman SL, Hoshida Y Hepatic stellate cells as key target in liver fibrosis. 2017; 14: 397-411.

[5] Skwarska A, Konopleva M (2023). BCL-xL Targeting to Induce Apoptosis and to Eliminate Chemotherapy-Induced Senescent Tumor Cells: From Navitoclax to Platelet-Sparing BCL-xL PROTACs. Cancer Res.

[6] Sun Z, Liang C, Zhao Y, Wu J, Wen L, Liu X (2025). aHSCs-targeted bimetallic nanozymes and luteolin-loaded liposomes: Synergistic reversal of liver fibrosis via antioxidant, cellular senescence, and cellular apoptotic mechanisms. Mater Today Bio.

[7] Chen Y, Choi SS, Michelotti GA, Chan IS, Swiderska M, Krüger L Hedgehog controls hepatic stellate cell fate by regulating metabolism. 2012; 56: 359-67.

[8] Du K, Hyun J, Premont RT, Choi SS, Michelotti GA, Swiderska-Syn M Hedgehog-YAP signaling pathway regulates glutaminolysis to control activation of hepatic stellate cells. 2018; 14: 276-88.

[9] Trivedi P, Wang S, Friedman SL (2021). The Power of Plasticity-Metabolic Regulation of Hepatic Stellate Cells. Cell Metab.

[10] Xie N, Tan Z, Banerjee S, Cui H, Ge J, Liu RM (2015). Glycolytic Reprogramming in Myofibroblast Differentiation and Lung Fibrosis. Am J Respir Crit Care Med.

[11] Zhang D, Tang Z, Huang H, Zhou G, Cui C, Weng Y (2019). Metabolic regulation of gene expression by histone lactylation. Nature.

[12] Amann T, Bataille F, Spruss T, Mühlbauer M, Gäbele E, Schölmerich J (2009). Activated hepatic stellate cells promote tumorigenicity of hepatocellular carcinoma. Cancer Sci.

[13] Roberts DJ, Miyamoto S (2015). Hexokinase II integrates energy metabolism and cellular protection: Akting on mitochondria and TORCing to autophagy. Cell Death Differ.

[14] Tsuchida T, Friedman SL (2017). Mechanisms of hepatic stellate cell activation. Nat Rev Gastroenterol Hepatol.

[15] Gao R, Tang H, Mao J (2023). Programmed Cell Death in Liver Fibrosis. J Inflamm Res.

[16] Linkermann A, Green DR Necroptosis. 2014; 370: 455-65.

[17] Jia Y, Gao L, Yang X, Zhang F, Chen A, Wang S (2020). Blockade of periostin-dependent migration and adhesion by curcumol via inhibition of nuclear factor kappa B signaling in hepatic stellate cells. Toxicology.

[18] Zheng Y, Wang L, Wang J, Zhao T, Wang J (2024). Modulation of the HIF-1α-NCOA4-FTH1 Signaling Axis Regulating Ferroptosis-induced Hepatic Stellate Cell Senescence to Explore the Anti-hepatic Fibrosis Mechanism of Curcumol. Curr Med Chem.

[19] Sun S, Li Z, Huan S, Kai J, Xia S, Su Y (2022). Modification of lysine deacetylation regulates curcumol-induced necroptosis through autophagy in hepatic stellate cells. Phytother Res.

[20] Rho H, Terry AR, Chronis C, Hay N (2023). Hexokinase 2-mediated gene expression via histone lactylation is required for hepatic stellate cell activation and liver fibrosis. Cell Metab.

[21] Friedman SL (2008). Hepatic stellate cells: Protean, multifunctional, and enigmatic cells of the liver. Physiological Reviews.

[22] Lin HJ, Tseng CP, Lin CF, Liao MH, Chen CM, Kao ST (2011). A Chinese Herbal Decoction, Modified Yi Guan Jian, Induces Apoptosis in Hepatic Stellate Cells through an ROS-Mediated Mitochondrial/Caspase Pathway. Evid Based Complement Alternat Med.

[23] Tungcharoen P, Wattanapiromsakul C, Tansakul P, Nakamura S, Matsuda H, Tewtrakul S (2018). Antiinflammation constituents from Curcuma zedoaroides. Phytotherapy Research.

[24] Li H, Sureda A, Devkota HP, Pittalà V, Barreca D, Silva AS (2020). Curcumin, the golden spice in treating cardiovascular diseases. Biotechnology Advances.

[25] Li Y, Li M, Mao J, Guo Q, Zhu W, Fu R (2024). The processing mechanism of vinegar-processed Curcumae Rhizome enhances anti hepatic fibrotic effects through regulation of PI3K/Akt/mTOR signaling pathway. Phytomedicine.

[26] Hao M, Ji D, Li L, Su L, Gu W, Gu L (2018). Mechanism of Curcuma wenyujin Rhizoma on Acute Blood Stasis in Rats Based on a UPLC-Q/TOF-MS Metabolomics and Network Approach. Molecules.

[27] Hosseini A, Hosseinzadeh H (2018). Antidotal or protective effects of Curcuma longa (turmeric) and its active ingredient, curcumin, against natural and chemical toxicities: A review. Biomedicine & Pharmacotherapy.

[28] Li Y, Zhou Y, Xia S, Chen L, Yang T, Zhao D (2023). Blockade of KLF5/LDH—A feedback loop contributes to Curcumol inhibition of sinusoidal endothelial cell glycolysis and mitigation of liver fibrosis. Phytomedicine.

[29] Yang J, Luo L, Zhao C (2022). A Positive Feedback Loop between Inactive VHL-Triggered Histone Lactylation and PDGFRβ Signaling Drives Clear Cell Renal Cell Carcinoma Progression. International Journal of Biological Sciences.

[30] Rabinowitz JD, Enerbäck S (2020). Lactate: the ugly duckling of energy metabolism. Nat Metab.

[31] Xie B, Zhang M, Li J, Cui J, Zhang P, Liu F (2024). KAT8-catalyzed lactylation promotes eEF1A2-mediated protein synthesis and colorectal carcinogenesis. Proceedings of the National Academy of Sciences.

[32] Jia M, Yue X, Sun W, Zhou Q, Chang C, Gong W (2023). ULK1-mediated metabolic reprogramming regulates Vps34 lipid kinase activity by its lactylation. Science Advances.

[33] Li L, Lei Q, Zhen Y, Cao L, Dong Y, Liu X (2024). Lactate Dehydrogenase Inhibition Protects against Hepatic Fibrosis by Regulating Metabolic Reprogramming of Hepatic Stellate Cells. J Agric Food Chem.

[34] Zhou Y, Yan J, Huang H, Liu L, Ren L, Hu J (2024). The m(6)A reader IGF2BP2 regulates glycolytic metabolism and mediates histone lactylation to enhance hepatic stellate cell activation and liver fibrosis. Cell Death Dis.

[35] Zhai X, Qiao H, Guan W, Li Z, Cheng Y, Jia X (2015). Curcumin regulates peroxisome proliferator-activated receptor-γ coactivator-1α expression by AMPK pathway in hepatic stellate cells *in vitro*. Eur J Pharmacol.

[36] Zhang W, Wang Z, Chen T (2011). Curcumol induces apoptosis via caspases-independent mitochondrial pathway in human lung adenocarcinoma ASTC-a-1 cells. Med Oncol.

[37] Guo X, Zheng B, Wang J, Zhao T, Zheng Y (2024). Exploring the mechanism of action of Chinese medicine in regulating liver fibrosis based on the alteration of glucose metabolic pathways. Phytother Res.

[38] Zhang Z, Guo M, Li Y, Shen M, Kong D, Shao J (2020). RNA-binding protein ZFP36/TTP protects against ferroptosis by regulating autophagy signaling pathway in hepatic stellate cells. Autophagy.

[39] Mederacke I, Dapito DH, Affò S, Uchinami H, Schwabe RF (2015). High-yield and high-purity isolation of hepatic stellate cells from normal and fibrotic mouse livers. Nat Protoc.

[40] Schäfer S, Zerbe O, Gressner AM (1987). The synthesis of proteoglycans in fat-storing cells of rat liver. Hepatology.

[41] Bourgognon M, Klippstein R, Al-Jamal KT Kupffer Cell Isolation for Nanoparticle Toxicity Testing. J Vis Exp. 2015: e52989.

[42] Pakkir Shah AK, Walter A, Ottosson F, Russo F, Navarro-Diaz M, Boldt J (2025). Statistical analysis of feature-based molecular networking results from non-targeted metabolomics data. Nature Protocols.

