# Supplementary material for: Curcumol Induces Necroptosis of Hepatic Stellate Cells by Targeting KAT8 to Suppress HK2 Lactylation and Promote HUWE1-Dependent Ubiquitination
Source: Int J Biol Sci. 2026 Jan 14;22(3):1648–73. doi: 10.7150/ijbs.125009 (PMC12839083; doi:10.7150/ijbs.125009)
Supplement: Supplementary file 1 — Supplementary figures. [file ijbsv22p1648s1.pdf]

Supplementary Figure S1

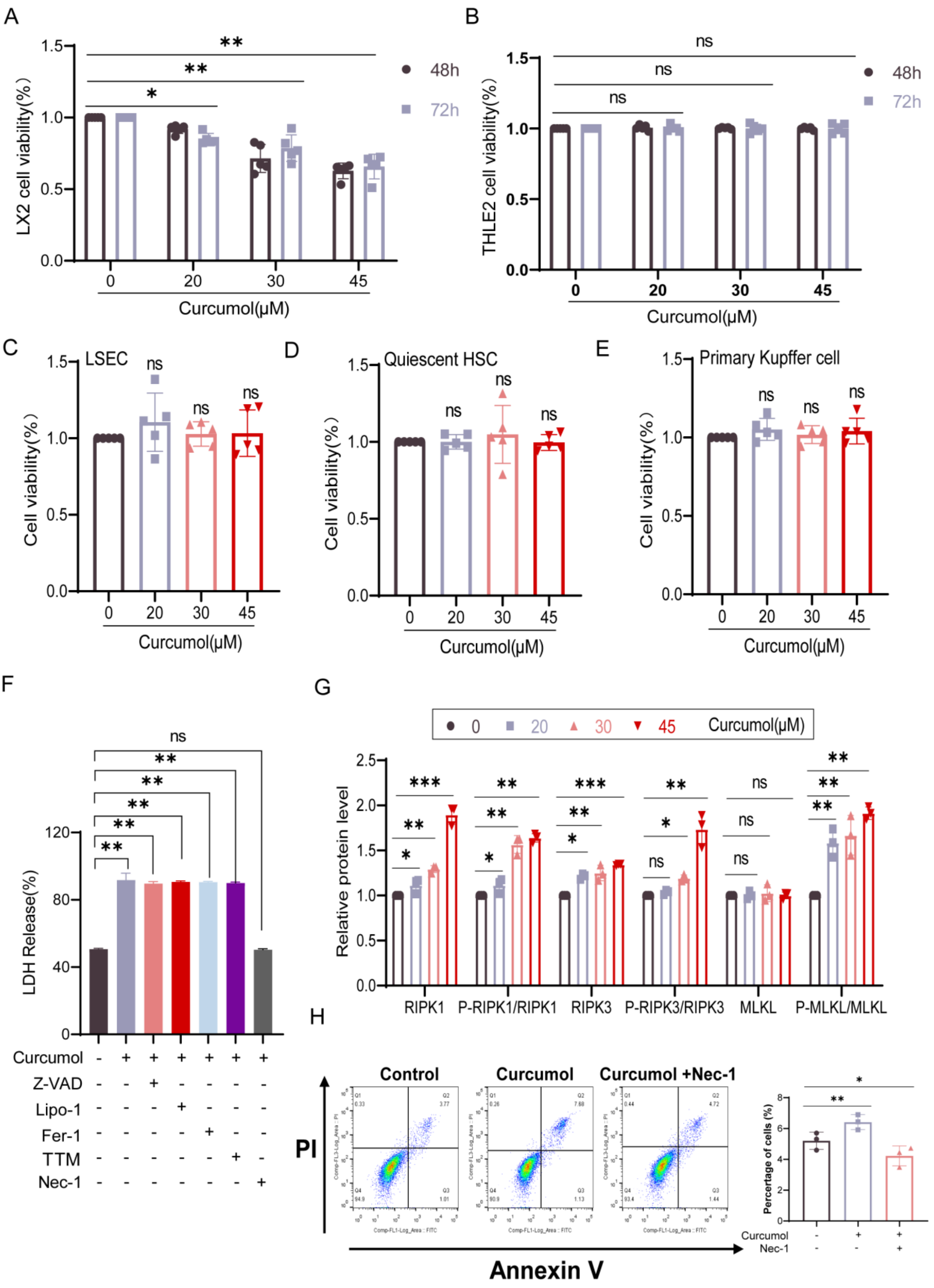

Supplementary Figure S1

A-B. Cell viability was measured in LX2 cells, human normal liver cells Thle-2 after treatment with Curcumol (0–45 μM) for 48h,72h (CCK-8 assay, n = 5).

C-E. Cell viability was measured in LSEC,Quiescent primary mouse HSCs and primary

mouse Kupffer cells after treatment with Curcumol (0–45 μM) for 24h (CCK-8 assay, n = 5).

F. Detection of LDH release levels in LX2 cells treated with different concentrations of Curcumol (0–45 μM) for 24 h (n = 5); simultaneous comparison of the effects of Nec-1 (50 μM), Z-VAD (20μM), Lipo-1 (0.5μM), Fer-1 (0.5μM) and TTM (20μM) treatment on curcumol (30 μM)-induced LDH release, with quantitative analysis (n = 5).

G.Quantitative data of Figure 2I protein immunoblotting(n = 3).

H. Under the same treatment conditions, the proportion of necroptotic cells was detected by flow cytometry, and the results were quantitatively analyzed (n = 3).Data are shown as mean ± SD, and statistical differences were analyzed by one-way ANOVA. ns indicates no significance. *$p < 0.05$, **$p < 0.01$, ***$p < 0.001$.

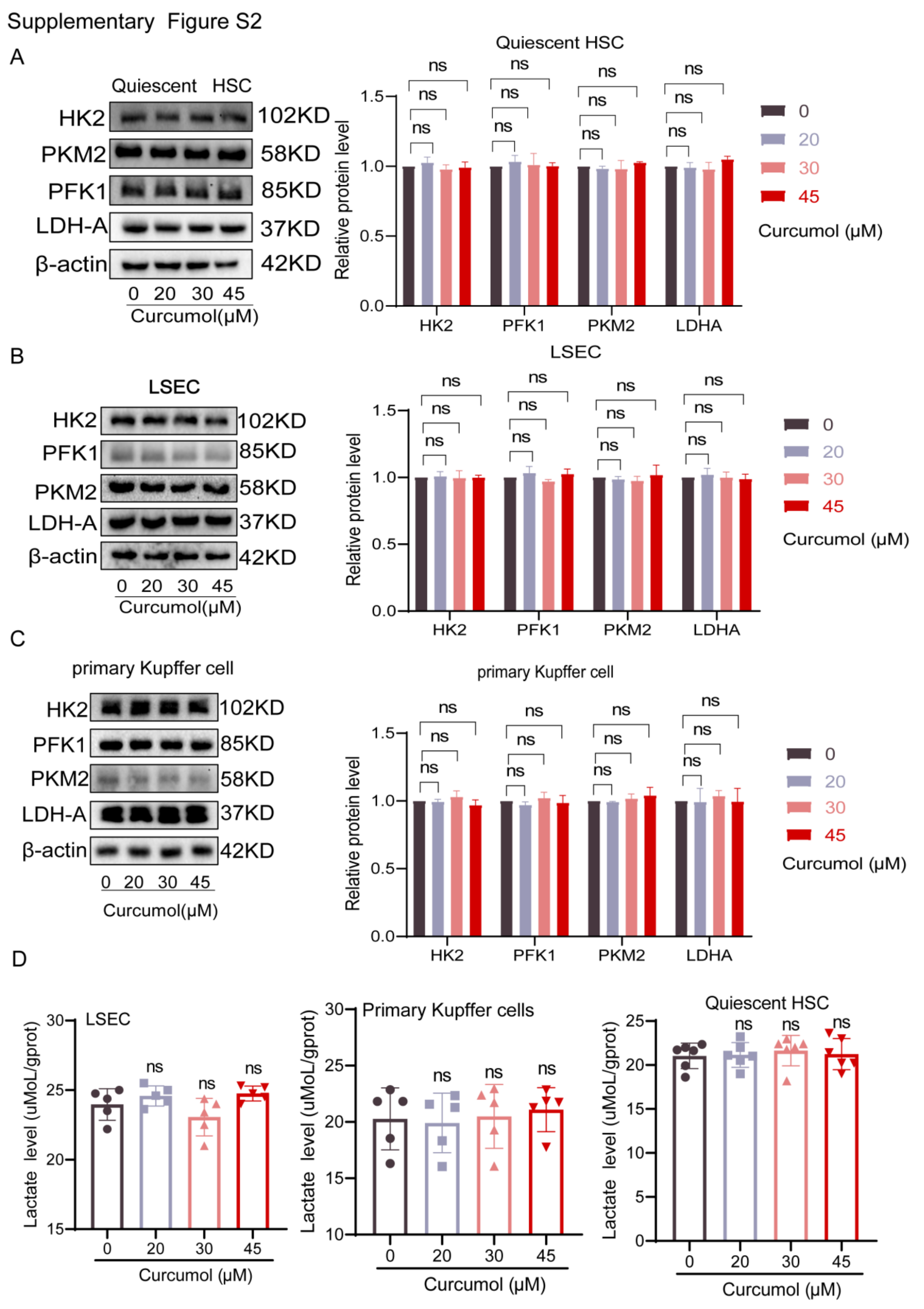

Supplementary Figure S2

A-C. Western blot analysis of HK2, PKM2, PFK1, and LDHA protein expression in Quiescent HSCs,LSEC and Primary Kupffer cells treated with Curcumol (0–45 μM) for 24 h, with densitometric quantification (n = 3).

D. Lactate levels in Quiescent HSC,LSEC and primary Kupffer cells treated with Curcumol (0–45 μM) for 24 h, measured using a lactate assay kit (n = 5).Data are shown as mean ± SD, and statistical differences were analyzed by one-way ANOVA. ns indicates no significance.*$p < 0.05$, **$p < 0.01$, ***$p < 0.001$.

Supplementary Figure S3

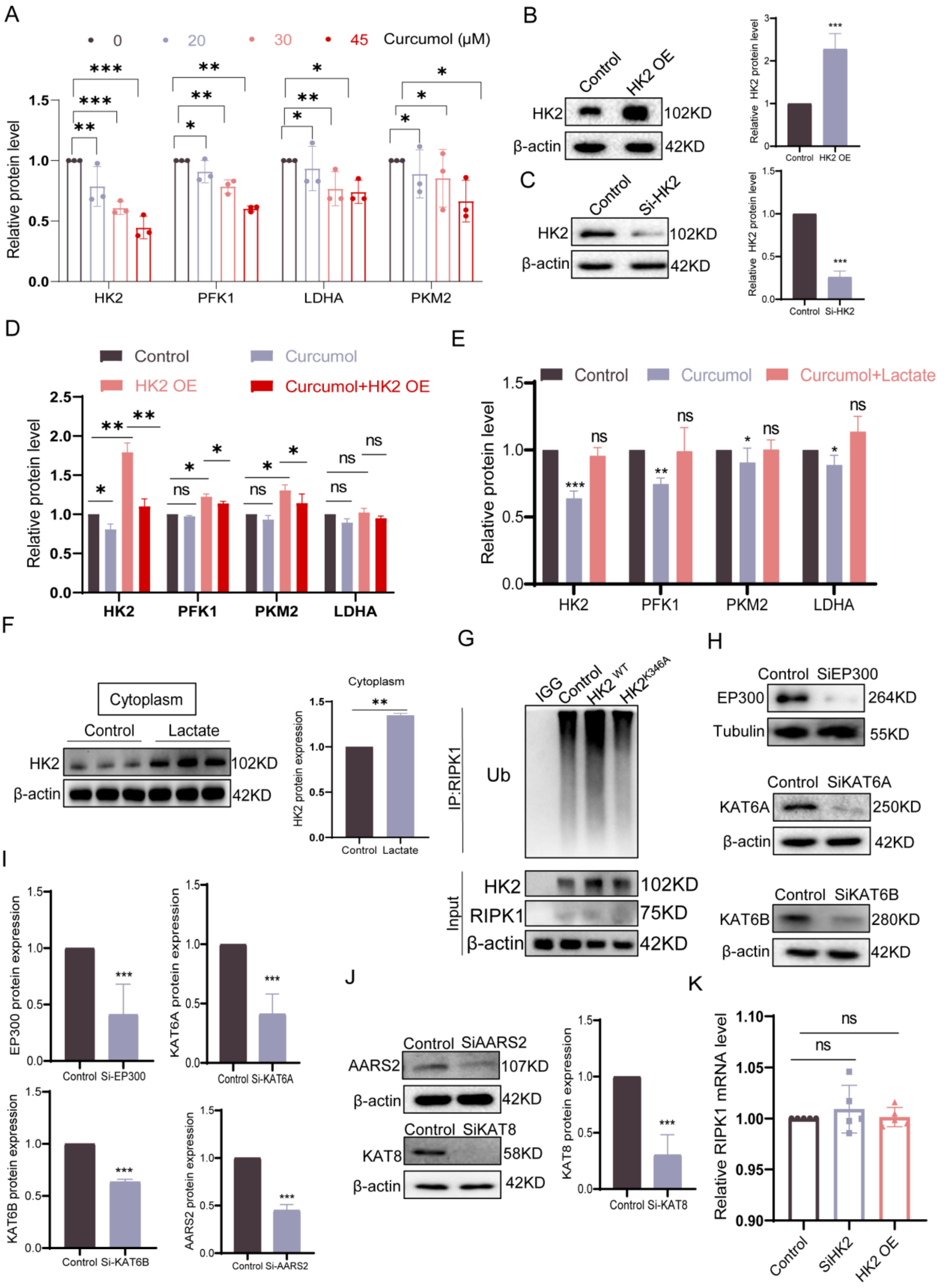

Supplementary Figure S3

A.Quantitative data of Figure 3C protein Western blot($n = 3$).

B-C. Verify the interference or overexpression efficiency of HK2 through Western blot and quantify it through grayscale analysis($n = 3$)

D. Quantitative data of Figure 3K protein Western blot($n = 3$).

E. Quantitative data of Figure 3Q protein Western blot($n = 3$).

F. Western blot analysis of HK2 expression in cytoplasmic fractions of LX2 cells treated with sodium lactate (10 mM) for 24 h, with densitometric quantification($n=3$).

G. CO-IP analysis of RIPK1 ubiquitination in LX2 cells transfected with HK2 WT and HK2 K346A).

H-J. Verify the interference or overexpression efficiency of EP300、KAT6A、KAT6B、KAT8 and AARS2 through Western blot and quantify it through grayscale analysis($n = 3$).

K.RT-qPCR analysis of RIPK1 mRNA expression in LX2 cells treated with increasing concentrations of SiHK2 and HK2 OE for 24 h ($n = 5$).Data are shown as mean ± SD, and statistical differences were analyzed by one-way ANOVA. ns indicates no significance.*$p < 0.05$, **$p < 0.01$, ***$p < 0.001$.

Supplementary Figure S4

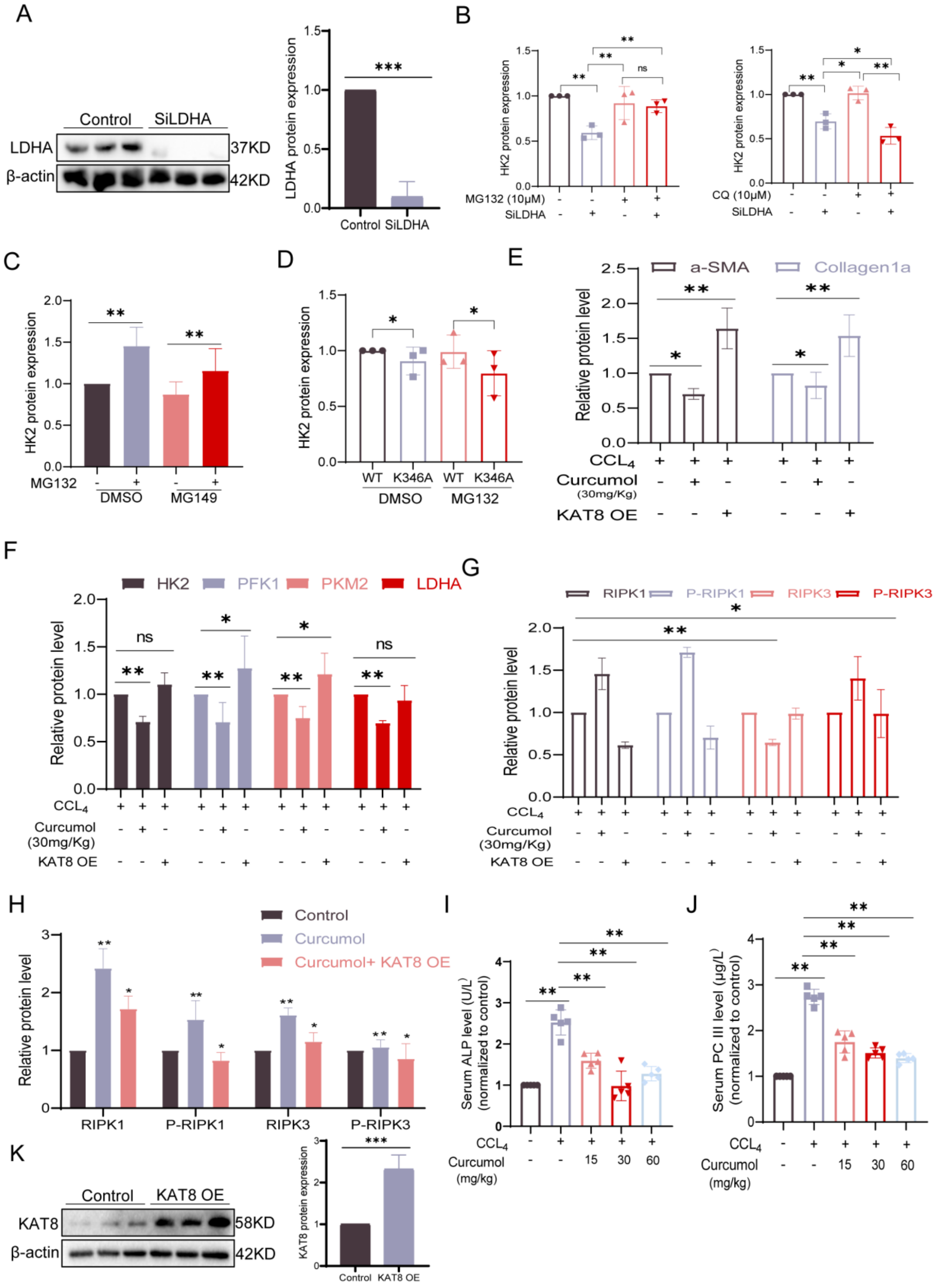

Supplementary Figure S4

A.Verify the interference efficiency of LDHA through Western blot and quantify it through grayscale analysis(n = 3).

B.Quantitative data of Figure 7B protein immunoblotting(n = 3).

C.Quantitative data of Figure 7F protein immunoblotting(n = 3).

D.Quantitative data of Figure 7D protein immunoblotting(n = 3).

E-F. Quantitative data of Figure 9G protein immunoblotting(n = 3).

H. Quantitative data of Figure 9H protein immunoblotting(n = 3).

I-J. Serum levels of ALP and PCIII in CCl_4-induced fibrotic mice treated with vehicle, Curcumol, or KAT8 OE + Curcumol, measured by biochemical assays (n = 5).

K. Verify the overexpression efficiency of KAT8 through Western blot and quantify it through grayscale analysis(n = 3).Data are shown as mean ± SD, and statistical differences were analyzed by one-way ANOVA. ns indicates no significance. *$p < 0.05$, **$p < 0.01$, ***$p < 0.001$.

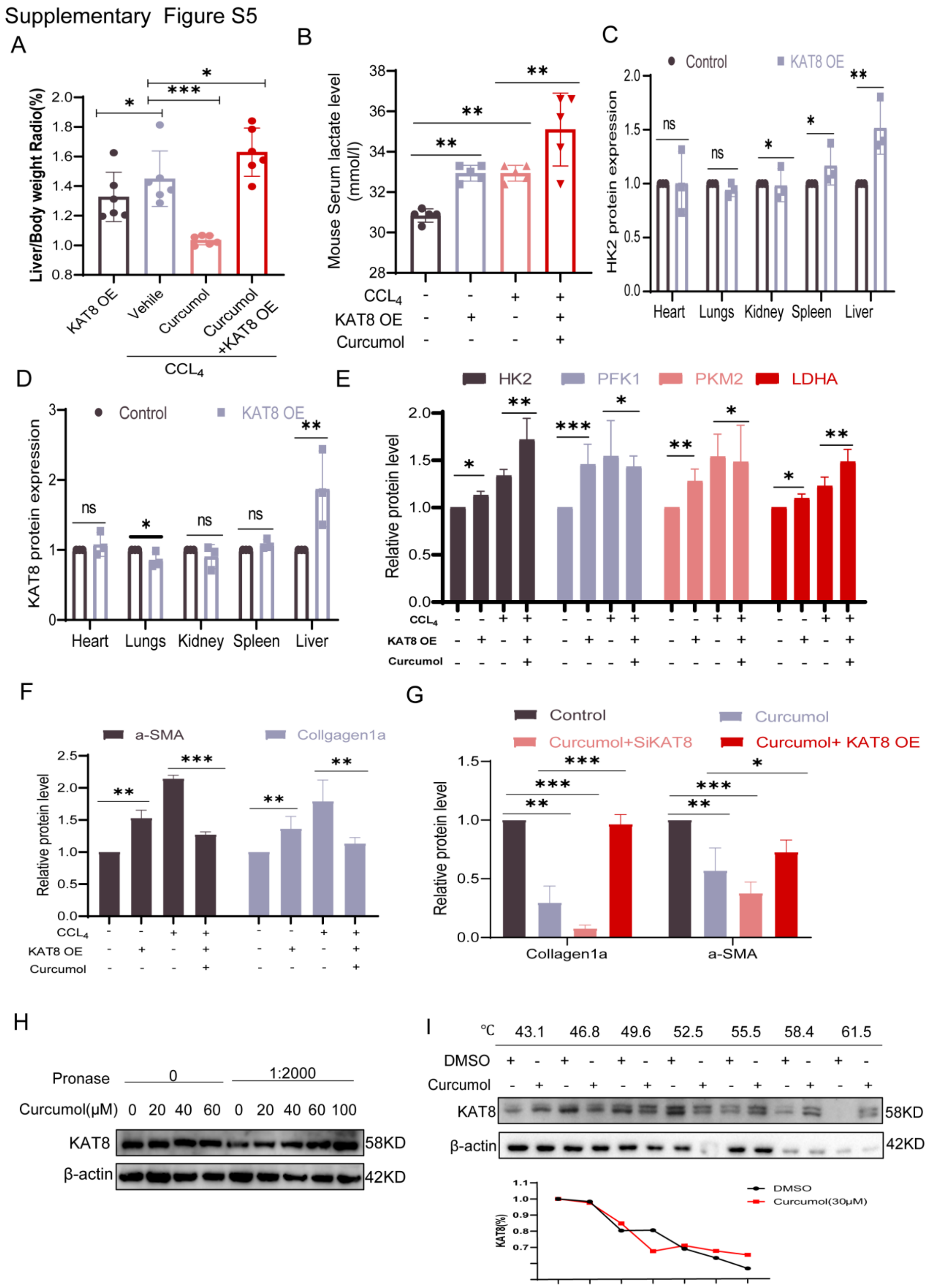

Supplementary Figure S5

A.Liver-to-body weight ratios were calculated for CCl4 model, Curcumol 30mg/kg-treated, and KAT8 OE + Curcumol 30mg/kg-treated mice (n = 5).

B.Serum lactate levels were measured using a lactate detection kit in CCl4-induced

fibrotic mice following Curcumol or KAT8 OE + Curcumol （30mg/kg)treatment (n = 5).

C-D.Quantitative data of Figure 9F protein immunoblotting(n = 3).

E-F.Quantitative data of Figure 9G protein immunoblotting(n = 3).

G.Quantitative data of Figure 9H protein immunoblotting(n = 3).

H. LX2 cells were digested with several dosages of protease with or without various concentrations of Curcumol, then KAT8 protein levels were assayed by western blot.

I.CETSA analysis of Curcumol (30 μM, 24 h) binding stability with KAT8 protein in LX2 cells (n = 3). Data are shown as mean ± SD, and statistical differences were analyzed by one-way ANOVA. ns indicates no significance. *$p < 0.05$, **$p < 0.01$, ***$p < 0.001$.

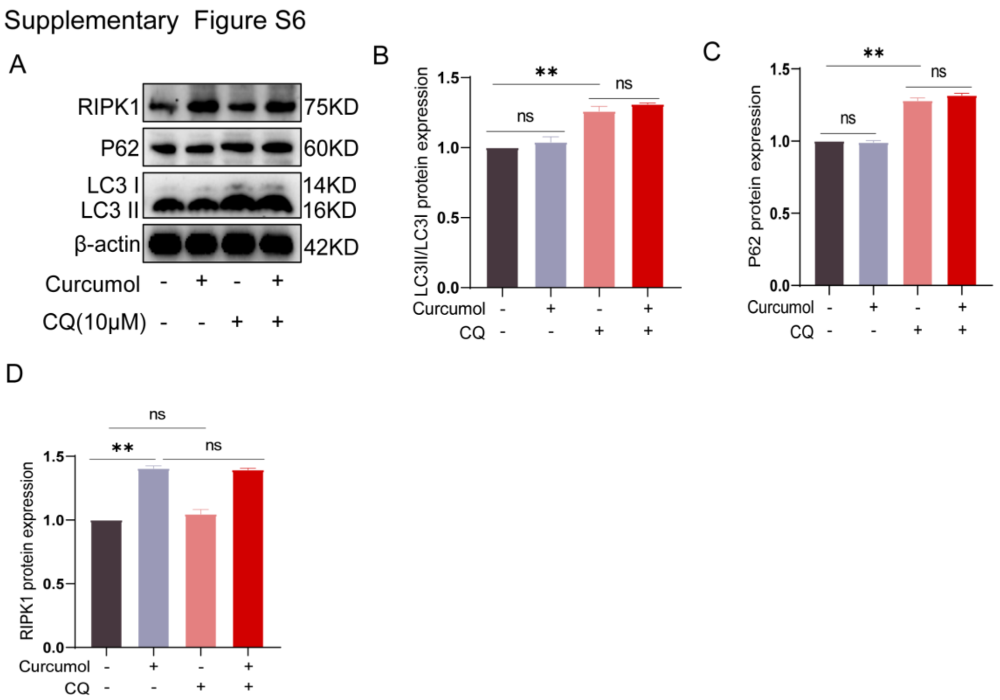

Supplementary Figure S6

A-D.Western blot analysis of LC3B, P62 and RIPK1 expression in LX2 cells treated with Curcumol (30 μM) and CQ (30 μM) for 24 h, with densitometric quantification (n = 3).
